# Loss of Diphthamide Increases DNA Replication Stress in Mammalian Cells by Modulating the Translation of RRM1

**DOI:** 10.1021/acscentsci.4c00967

**Published:** 2024-09-06

**Authors:** Jiaqi Zhao, Byunghyun Ahn, Hening Lin

**Affiliations:** a Department of Chemistry and Chemical Biology, Cornell University, Ithaca, New York 14853, United States; b Department of Molecular Biology and Genetics, Cornell University, Ithaca, New York 14853, United States; c Howard Hughes Medical Institute, Cornell University, Ithaca, New York 14853, United States

## Abstract

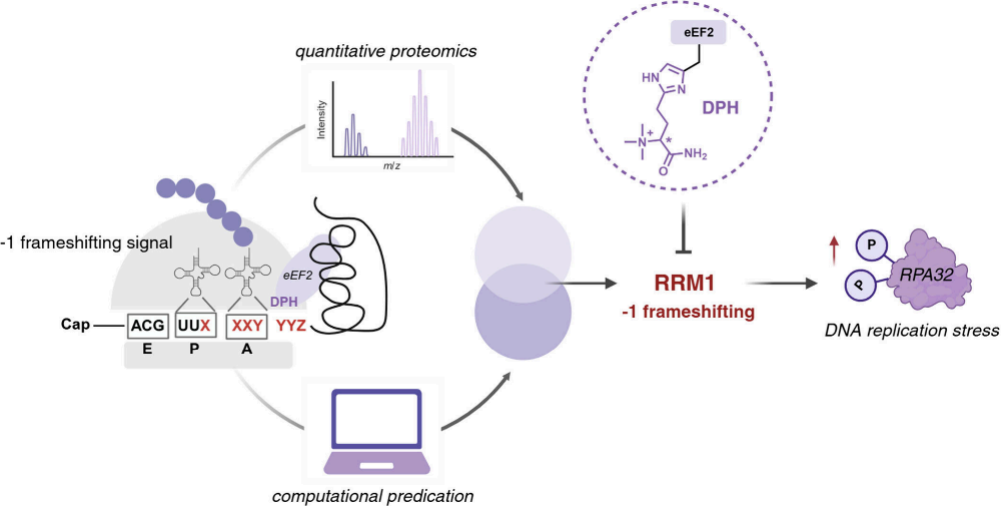

Diphthamide (DPH) is a highly conserved post-translational modification exclusively present in eukaryotic translation elongation factor 2 (eEF2), with its loss leading to embryonic lethality in mice and developmental disorders in humans. In this study, we unveil the role of diphthamide in mammalian cell DNA damage stress, with a particular emphasis on DNA replication stress. We developed a systematic strategy to identify human proteins affected by diphthamide with a combination of computational profiling and quantitative proteomics. Through this approach, we determine that the translation of RRM1 is modulated by diphthamide via −1 frameshifting. Importantly, our results reveal that the dysregulation of RRM1 translation in DPH-deficient cells is causally linked to elevated DNA replication stress. These findings provide a potential explanation for how diphthamide deficiency leads to cancer and developmental defects in humans.

## Abstract 2

Integration of computational and proteomic methods reveals that the loss of diphthamide leads to DNA replication stress in mammalian cells by modulating RRM1 translation.

## Introduction

DNA damage stress has long been identified as a hallmark of various physiological and pathological conditions.^1−3^ Among the diverse types of DNA damage, DNA replication stress emerges as a critical contributor to genomic instability.^4^ During DNA replication, challenges such as DNA lesions and replication fork stalling can generate single-stranded DNA (ssDNA) regions and DNA double-stranded breaks (DSBs). These replication-associated DNA lesions trigger intricate cellular responses to preserve genomic integrity, including activating DNA damage response pathways and inducing cell cycle checkpoints.^4^ Dysregulation of these pathways or failure to resolve replication stress can result in the accumulation of mutations, chromosomal aberrations, and carcinogenesis.^2,3^

Ribonucleotide reductase (RNR) is a rate-limiting enzyme for *de novo* deoxyribonucleoside triphosphate (dNTP) production, playing a central role in maintaining genome integrity.^5^ RNR comprises two subunits, RRM1 and RRM2, with RRM1 being ubiquitously expressed and RRM2 being cell-cycle dependently expressed.^6^ Emerging evidence suggests that dysregulation of RRM1 expression^6^ or activity^7^ is closely associated with DNA replication stress and genomic instability. Moreover, aberrant RRM1 expression has been implicated in various human cancers and is considered a potential therapeutic target for cancer treatment.^5−7^ Therefore, understanding the intricate interplay between RRM1 and DNA replication stress is crucial to unraveling carcinogenesis mechanisms.

**Figure 1:**
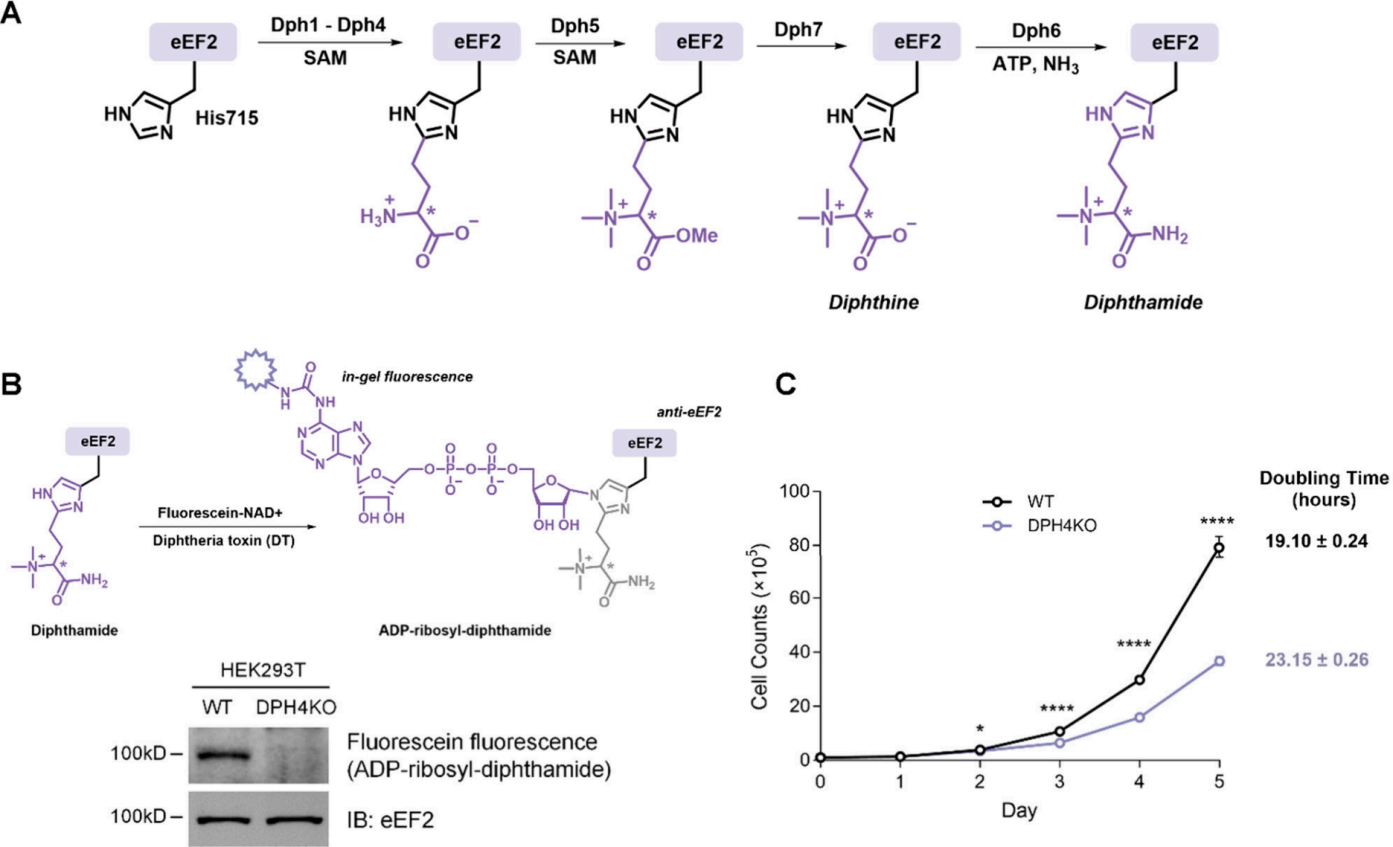
Diphthamide deficiency causes human cell line growth defects. (A) Structure and the biosynthetic pathway of diphthamide. (B) Verification of the diphthamide deficiency of HEK293T DPH4KO cells via diphtheria toxin-mediated ADP-ribosylation reaction that led to the fluorescent labeling of diphthamide. The diphthamide modification level was assessed by in-gel fluorescein fluorescence. The total eEF2 protein level was assessed by Western blotting. (C) Growth curve and cell doubling time of HEK293T WT and DPH4KO cells. Data with error bars are mean ± s.d. *P* values are determined by the unpaired Welch’s *t* test, *P < 0.05; ****P < 0.0001.

Diphthamide is a unique post-translational modification (PTM) whose biosynthesis requires four steps involving seven diphthamide biosynthesis genes (DPH1–7).^8^ In the initial step, a C–C bond is formed between the imidazole-C2 atom of His715 in eukaryotic elongation factor 2 (eEF2) and a 3-amino-3-carboxypropyl (ACP) group from *S*-adenosylmethionine (SAM) through a noncanonical radical SAM reaction. This transfer is facilitated by a protein complex formed by Dph1, Dph2, and Dph3, and it also requires Dph4. Following this, in the second step, Dph5 catalyzes the trimethylation of the amino group and the methylation of the carboxyl group of the ACP intermediate. Subsequently, Dph7 restores the carboxyl group through demethylation to yield the diphthine intermediate. Lastly, Dph6 utilizes ATP and ammonium to carry out the amidation of the carboxyl group to generate diphthamide (Figure 1A).^8^ Despite its chemically challenging biosynthetic pathway, diphthamide is exclusively found in one protein, eEF2,^9^ and evolutionarily conserved in most eukaryotes.^10^

Diphthamide was originally identified as the molecular target of diphtheria toxin (DT) from *Corynebacterium diphtheriae*, which catalyzes the NAD^+^-dependent ADP-ribosylation of diphthamide and hence inhibits global translation elongation.^11^ The evolutionary conservation of diphthamide and its elaborate biosynthesis in eukaryotes suggest its functional importance. Aligning with the essential role of eEF2 as a GTPase in the elongation step of translation,^12,13^ diphthamide has been shown to ensure translational fidelity by preventing the −1 frameshifting event during protein translation in yeast, plants, and mammalian cells.^10,14−20^ Recent structural studies suggested that diphthamide may prevent translational “slippage” through binding to mRNA during translocation.^15^ Since diphthamide plays a crucial role in translational fidelity, its deficiency can have substantial phenotypic consequences on both the cellular and organismal levels. Many critical regulatory and stress signaling pathways for development require diphthamide-modified eEF2 for proper translation.^21−23^ Loss of diphthamide biosynthesis genes can be lethal in mice,^14,24,25^ and mutations in these genes cause human developmental defects^26^ and carcinogenesis.^24,27^ However, through which protein targets diphthamide deficiency causes these phenotypes has not been elucidated.

Here, we unveil a role of diphthamide in mammalian cell DNA damage stress with a particular emphasis on DNA replication stress. Employing a novel strategy, we identified diphthamide-affected protein candidates by combining computational profiling of the human transcriptome and quantitative proteomics. Through this approach, we demonstrated that the translation of RRM1 is modulated by diphthamide and that the dysregulation of RRM1 translation contributes to the observed DNA replication stress. Our results provide a potential explanation for the association between diphthamide deficiency and cancer.

## Results

### Diphthamide Deficiency Contributes to Mammalian Cell Growth Defects and DNA Replication Stress

To explore the biological function of diphthamide in human cells, we generated HEK293T DPH4 CRISPR-knockout (DPH4KO) cells as a model system (Figure S1). It is well-established that deleting either of the seven diphthamide biosynthesis genes (DPH1–7) will result in the loss of the diphthamide modification in eEF2.^8^ The modification level could be visualized via an *in vitro* diphtheria toxin-mediated ADP-ribosylation reaction relying on a fluorescent NAD^+^ analog as the substrate.^28^ Only in cells that contain eEF2-diphthamide could DT modify eEF2, which can be monitored via in-gel fluorescence and Western blotting. We confirmed the complete depletion of the diphthamide modification in eEF2 in DPH4KO cells via this assay (Figure 1B). While culturing the HEK293T wild-type (WT) and DPH4KO cells, we observed a notable difference in that DPH4KO cells exhibited slower cell growth and longer doubling times than WT cells (Figure 1C). This observation indicates that diphthamide deficiency leads to human cell line growth defects.

**Figure 2:**
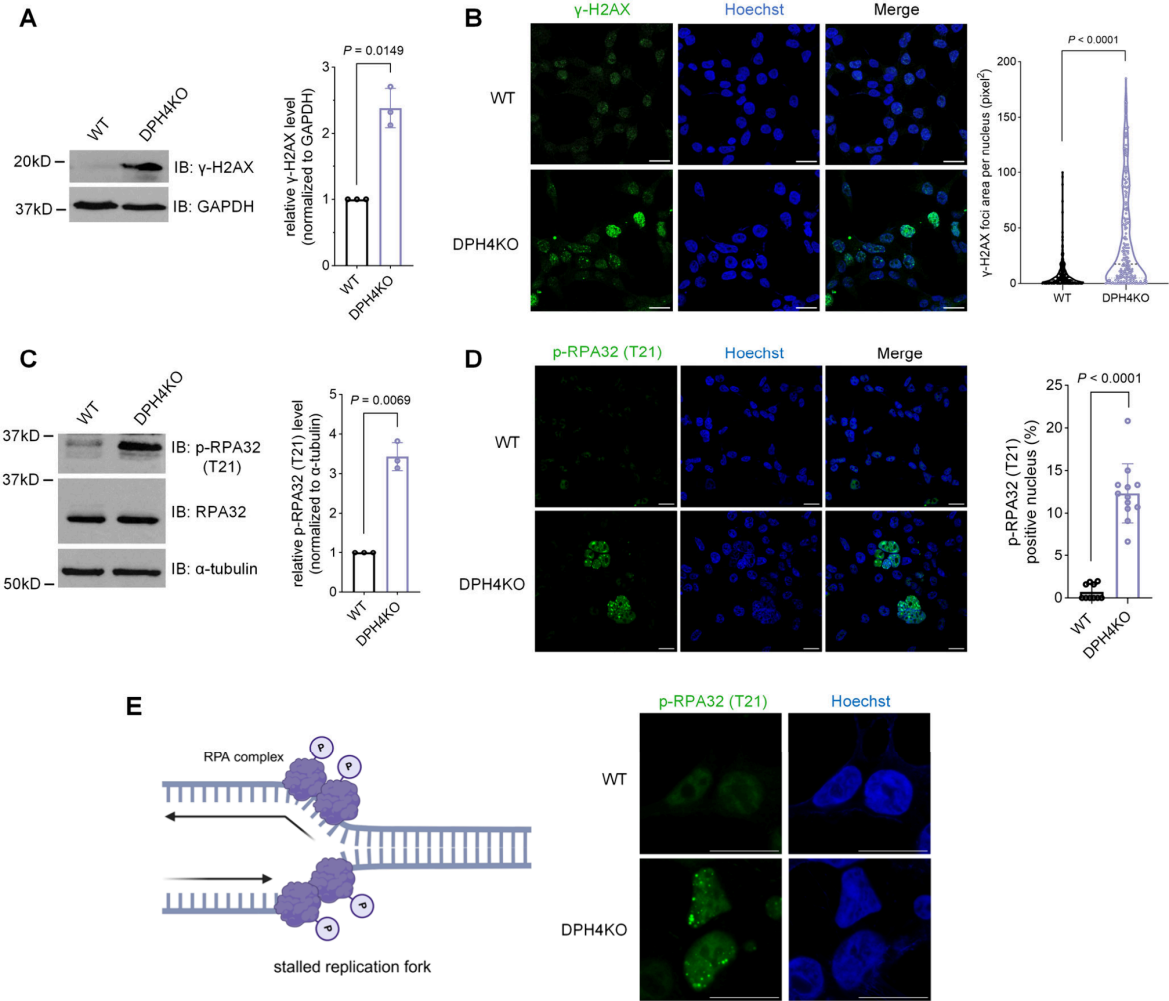
Diphthamide deficiency contributes to cellular DNA replication stress. (A) Immunoblot analysis and quantification of γ-H2AX levels in HEK293T WT and DPH4KO cells under normal growth conditions. (B) Immunofluorescence staining of γ-H2AX and quantification of γ-H2AX total area in the nuclei of HEK293T WT and DPH4KO cells under normal growth conditions. (C) Immunoblot analysis and quantification of p-RPA32 (Thr21) levels in HEK293T WT and DPH4KO cells under normal growth conditions. (D) Immunofluorescence analysis showing percentage of p-RPA32 (Thr21) nuclei in HEK293T WT and DPH4KO cells. (E) Cartoon illustration of DNA replication fork stalling and immunofluorescence analysis of p-RPA32 (Thr21) foci in HEK293T WT and DPH4KO cells. Representative data from three biologically independent experiments. Data with error bars are mean ± s.d. *P* values are determined by the unpaired Welch’s *t* test. Scale bars: 20 μm.

Accurate DNA replication and repair are fundamental processes for proper eukaryotic cell growth.^5^ Consequently, we investigated DNA damage stress levels in human cells lacking diphthamide modification. We assessed the level of DNA damage in cells by utilizing γ-H2AX as a biomarker for DNA damage and double-stranded breaks.^29^ Under normal growth conditions, HEK293T DPH4KO cells exhibited significantly higher levels of γ-H2AX, indicating an elevated DNA damage stress (Figure 2A). Immunofluorescence analysis also revealed increased γ-H2AX foci intensity in the nuclei of DPH4KO cells (Figure 2B), further supporting a higher level of DNA damage in cells lacking diphthamide modification.

Given the pivotal role of DNA replication in cell growth, we next investigated whether the depletion of diphthamide in human cells would specifically result in accumulated DNA replication stress. It has been reported that several N-terminal residues for RPA32, including Thr21, are phosphorylated in response to DNA replication stress.^30^ Immunoblot analysis revealed hyperphosphorylation of RPA32 in DPH4KO cells (Figure 2C), corroborated by an immunofluorescence assay showing a higher proportion of pRPA32-Thr21 positive nuclei in DPH4KO cells compared to WT cells (Figure 2D). Furthermore, since phosphorylated RPA32 at Thr21 binds to single-stranded DNA at the stalled replication fork,^30^ we assessed whether DPH deficiency would cause replication fork stalling. Foci of pRPA32-Thr21 were observed in the nuclei of DPH4KO cells but not in WT cells (Figure 2E), indicating the recruitment of pRPA32-Thr21 at the stalled replication forks in DPH4KO cells. Collectively, these results suggested that DPH depletion in cells caused DNA replication stress and fork stalling.

### Integration of Computational Analysis and Quantitative Proteomics to Identify Diphthamide Targets

We next aimed to elucidate the molecular mechanism underlying the observed DNA replication stress phenotype in DPH-deficient cells. Given the limited understanding of diphthamide functions, reported systematic strategies to identify its regulated protein targets are scarce. Previous studies have highlighted diphthamide’s crucial role in maintaining translation fidelity by suppressing −1 frameshifting.^15,17,31^ Therefore, it stands to reason that one of the primary mechanisms through which diphthamide functions is modulation of the protein translation process. We herein used a novel systematic approach to identify diphthamide-affected protein candidates in human cells.

**Figure 3:**
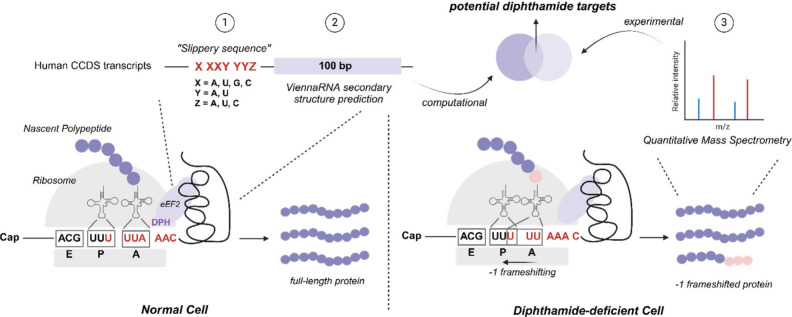
Integrating computational prediction and quantitative proteomics is a novel strategy for identifying diphthamide-affected protein candidates. Workflow for the identification of diphthamide-affected protein candidates: determine potential translation −1 frameshifting sites by ① genome-wide profiling of the CCDS database of human mRNA transcripts for “slippery sequence” and ② secondary structure and minimum free energy (MFE) prediction of the 100 bp mRNA following the slippery sequence by the ViennaRNA algorithm; experimentally identify diphthamide-affected proteins via ③ quantitative proteomics. Overlap of the computational and experimental analysis reveals potential diphthamide-affected protein candidates.

We hypothesized that as diphthamide deficiency increases −1 frameshifting rates, frameshifted proteins should occur in diphthamide-deficient cells, which subsequently manifests as protein level differences. To address this, we devised a two-step strategy: First, we employed transcriptome-wide computational prediction to identify which proteins are more prone to undergo −1 frameshifting during translation. Subsequently, we used quantitative proteomics to identify proteins that are altered in diphthamide-deficient cells. By integrating these two methods, we aimed to generate a list of genes whose protein level differences are more likely to be influenced by −1 frameshifting during translation and, hence, by diphthamide (Figure 3).

### Prediction of −1 Frameshifting Sites in the Human Transcriptome Based on the Slippery Sequence Motif

Following the proposed workflow (Figure 3), we first conducted genome-wide profiling of the CCDS database of human mRNA transcripts to find motifs as potential −1 frameshifting sites. It has been reported that diphthamide downregulates frameshifting of the HIV-programmed −1 frameshifting motif, characterized by “slippery sequences” followed by stable secondary structures without a spacer sequence.^14,15^ The “slippery sequences” feature of mRNA sequences typically follows the pattern X XXY YYZ (where X = any nucleotide, Y = A or U, Z = A, U, or C), particularly when positioned in front of a pseudoknot that may cause a pause in translation elongation.^32^

**Figure 4:**
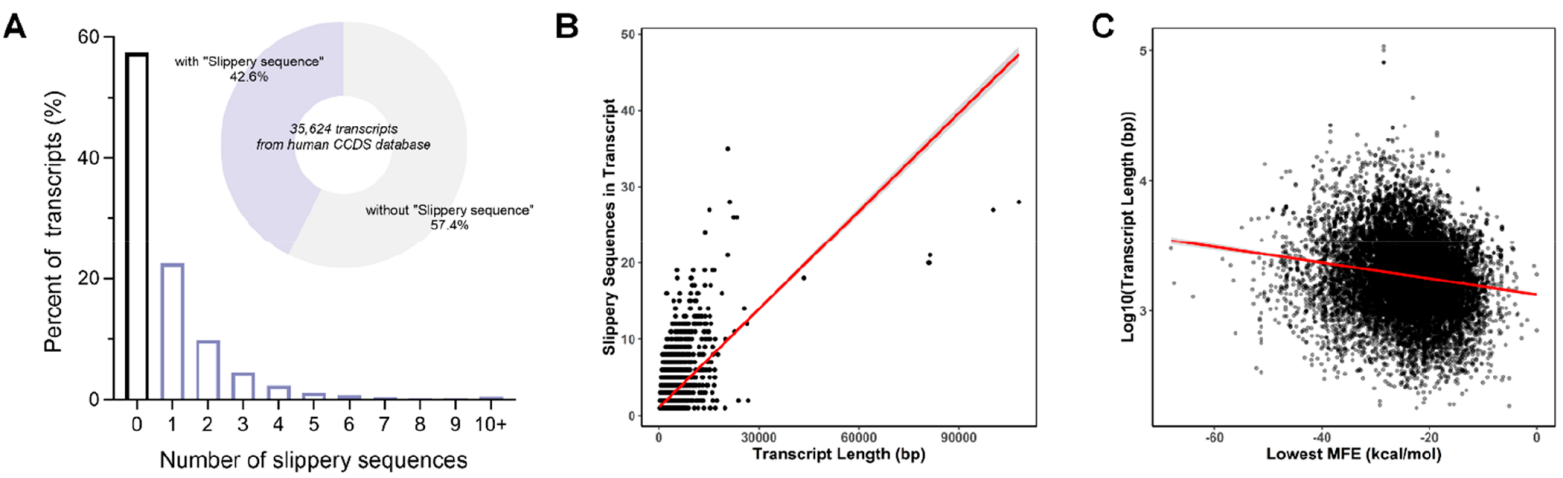
Prediction of −1 frameshifting sites in the human transcriptome based on the slippery sequence motif and minimum free energy (MFE). (A) Percentage of mRNA coding sequence transcripts containing different numbers of slippery sequences. (B) The number of slippery sequences in mRNA CDS transcripts plotted against the transcript length (bp). The red line is a linear regression fit to indicate the relationship between the number of slippery sequences in the transcript and the transcript size. (C) Transcript length is plotted against the lowest MFE (minMFE) values of the predicted slippery mRNA transcripts. The red line is a linear regression fit to indicate the relationship between transcript/protein size and MFE.

We computationally scanned the CCDS database of 35,624 mRNA coding sequence (CDS) transcripts to identify mRNA with slippery sequences. Out of 35,624 transcripts in the database, 15,173 contained at least one slippery sequence (Figure 4A). Generally, longer mRNAs contain more slippery sequences in the transcripts (Figure 4B). For the transcripts with at least one slippery sequence, we used *ViennaRNA* to calculate the minimum free energy (MFE) of the 100 bp mRNA following the slippery sequence as a predictor for the presence of potential pseudoknots that could promote frameshifting.^33,34^ For transcripts with multiple potential slippery sequence motifs, the lowest MFE value (“minMFE”) of all motifs was considered. Similar to the previous report that the minMFE of transcripts negatively correlates with the transcript size in the yeast genome,^35^ we observed lower minMFE in longer human transcripts (Figure 4C). Considering that transcripts with higher MFE values are less likely to form stable secondary structures, we chose the MFE cutoff value to be less than −10.0 kcal/mol. With this cutoff value, transcripts from 7980 genes were identified to have at least one potential −1 frameshifting site.

### SILAC Quantitative Proteomics Identifies Proteins Affected by Diphthamide

**Figure 5:**
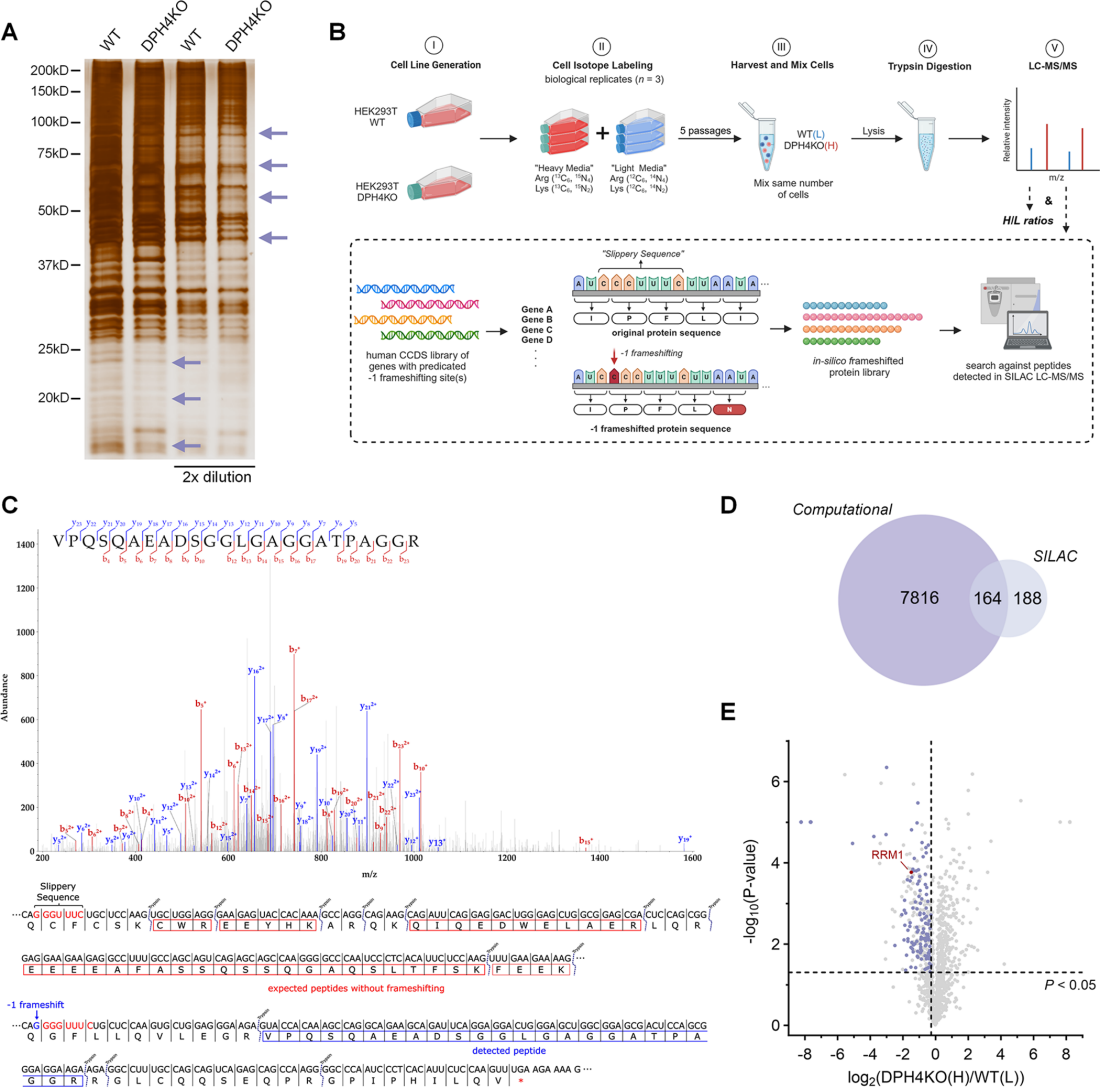
SILAC quantitative proteomics reveals proteome-wide differences associated with diphthamide deficiency. (A) Silver stain of HEK293T WT and DPH4KO cell lysates. (B) Workflow of designed SILAC proteomics experiment to quantitatively compare the proteome between HEK293T WT and DPH4KO cells. (C) MS/MS spectrum of the −1 frameshifted peptide VPQSQAEADSGGLGAGGATPAGGR detected in the HEK293T DPH4KO sample. Sequence alignment of RABGEF1 mRNA reading frames without or with the predicted −1 frameshifting event, showing the generation of VPQSQAEADSGGLGAGGATPAGGR peptide and a resulting nonsense mutation after −1 frameshifting. The slippery sequence is labeled in red. Theoretical trypsin-digested peptides from the original reading frame are highlighted in red boxes, and the detected −1 frameshifted peptide is highlighted in a blue box. (D) Venn diagram of the −1 frameshifting protein candidates from computational prediction and SILAC proteomics. 164 overlapping genes were identified by combining the two methods. (E) Volcano plot of SILAC quantitative proteomics. 164 identified targets are highlighted in purple, and RRM1 is highlighted in red.

We then sought to experimentally identify proteins affected by diphthamide modification in eEF2 (Figure 3). We hypothesized that the increased −1 frameshifting rates caused by DPH deficiency would result in decreased protein levels. To test this, we analyzed lysates from both HEK293T WT and DPH4KO cells. As expected, we observed differences in total protein levels between the two cell types (Figure 5A), with a significant number of proteins showing lower levels in DPH4KO cells than in WT cells (Figure 5A, indicated by arrows). To identify these proteins, we employed quantitative proteomics.

We chose Stable Isotope Labeling by Amino Acids in Cell Culture (SILAC) for its high resolution and accuracy.^36^ Furthermore, the metabolic labeling process in SILAC enabled us to mix equal numbers of cells, which better preserves the observed proteome-wide differences than combining the same amount of total proteins. We used HEK293T WT and DPH4KO cells grown in media supplemented with light- or heavy-labeled lysine and arginine, respectively. Equal numbers of light-labeled WT cells and heavy-labeled DPH4KO cells (WT(L)/DPH4KO(H)) were mixed. After lysis and trypsin digestion, the resulting peptides were subjected to LC-MS/MS analysis (Figure 5B).

We initially analyzed the proteomics results by quantifying detected peptides based on their heavy-to-light (H/L) ratios (Figure 5B and Table S3). To ensure the reliability of our findings, especially given that we might be looking at small changes in protein levels, we conducted the SILAC experiment with three biological replicates, and we only considered proteins with an H/L ratio cutoff of 0.83 or 1.5 and ratio-abundance *P*-value of less than 0.05. Notably, proteins exhibiting increased levels in DPH4KO cells and approximately 37 and 15 kDa in size were identified (Table S1), aligning with our silver stain analysis (Figure 5A). For proteins exhibiting decreased levels in DPH-deficient cells, our primary interest, this criterion yielded a list of 350 candidates.

### Frameshifted Peptides Were Identified in DPH-Deficient Cells Using an *in-Silico* −1 Frameshifted Protein Library

While the above analysis of the SILAC proteomics results allowed us to identify potential proteins affected by diphthamide, it does not directly identify the −1 frameshifted peptides generated in cells. Therefore, we sought to develop an approach to directly detect the frameshift events in cells without diphthamide using an *in-silico* library of −1 frameshifted proteins for LC-MS/MS analysis. To construct an *in-silico* library of −1 frameshifted human proteome based on the slippery sequence hypothesis, we employed the list of identified CCDS transcripts containing at least one slippery sequence motif and a minMFE less than −10 kcal/mol. These transcripts were subjected to an *in-silico* −1 frameshifting process based on the slippery sequence site, followed by translation into amino acid sequences until the first stop codon was encountered. If the −1 frameshifting event completely removed the stop codon in the original CDS region, the 3′-untranslated region (3′-UTR) was incorporated until a stop codon was reached. These newly generated hypothetical translated protein sequences constituted the −1 frameshifted library for the peptide search in LC-MS/MS analysis (Figure 5B). We found that more than 95% of the transcripts were predicted to result in a “truncated protein” after the −1 frameshifting event (Figure S2), which reassures our approach to assessing proteins with reduced levels through quantitative proteomics.

Trypsin-digested peptides from HEK239T WT and DPH4KO cells in the SILAC experiment were searched against this *in-silico* library. We set the false discovery rate (FDR) as 1% to ensure high confidence in peptide identification. One peptide, VPQSQAEADSGGLGAGGATPAGGR, was confidently identified exclusively in DPH4KO cells (Figure 5C). This peptide sequence is not documented in any reported human protein database but was found to match a −1 frameshifted transcript of RABGEF1 in our hypothetical library (Figure 5C). Given that this −1 frameshifting event also generates a nonsense mutation, resulting in preterminated proteins, we envisioned that the protein level of RABGEF1 could be affected in DPH-deficient cells. Immunoblot analysis indeed revealed a decrease of endogenous RABGEF1 protein level upon DPH4 knockout (Figure S3), consistent with the DPH-dependent −1 frameshifting event during RABGEF1 translation. Another peptide, ALPQLSDDIPFPSQLAR, a hypothetical −1 frameshifted peptide of the ERLEC1 protein, was also exclusively detected in DPH4KO cells (Figure S4). These findings provide direct evidence of −1 frameshifting events in DPH-deficient cells and serve as a proof-of-concept trial using the *in-silico* library to study −1 frameshifting events.

### Combining the *in-Silico* Prediction with SILAC Proteomics Identifies RRM1 as a Diphthamide Target

Our proteomics results indicated that a total of 352 proteins met the set H/L ratio cutoffs or were found in the *in-silico* frameshifted library. Of these, 164 overlapped with the list of genes computationally analyzed to contain potential −1 frameshifting sites with MFE less than −10 kcal/mol (Figure 5D and Table S4). To further explore the observed elevated DNA replication stress, we focused on proteins associated with DNA repair. Among these, ribonucleotide reductase regulatory subunit M1 (RRM1) particularly attracted our attention. The SILAC results revealed significant decreases in RRM1 protein abundance in all three replicates of DPH4KO cells (Figure 5E). Moreover, RRM1’s ubiquitous and high expression levels across tissue types^6^ further underscored its importance, prompting us to select it for additional biochemical validation.

**Figure 6:**
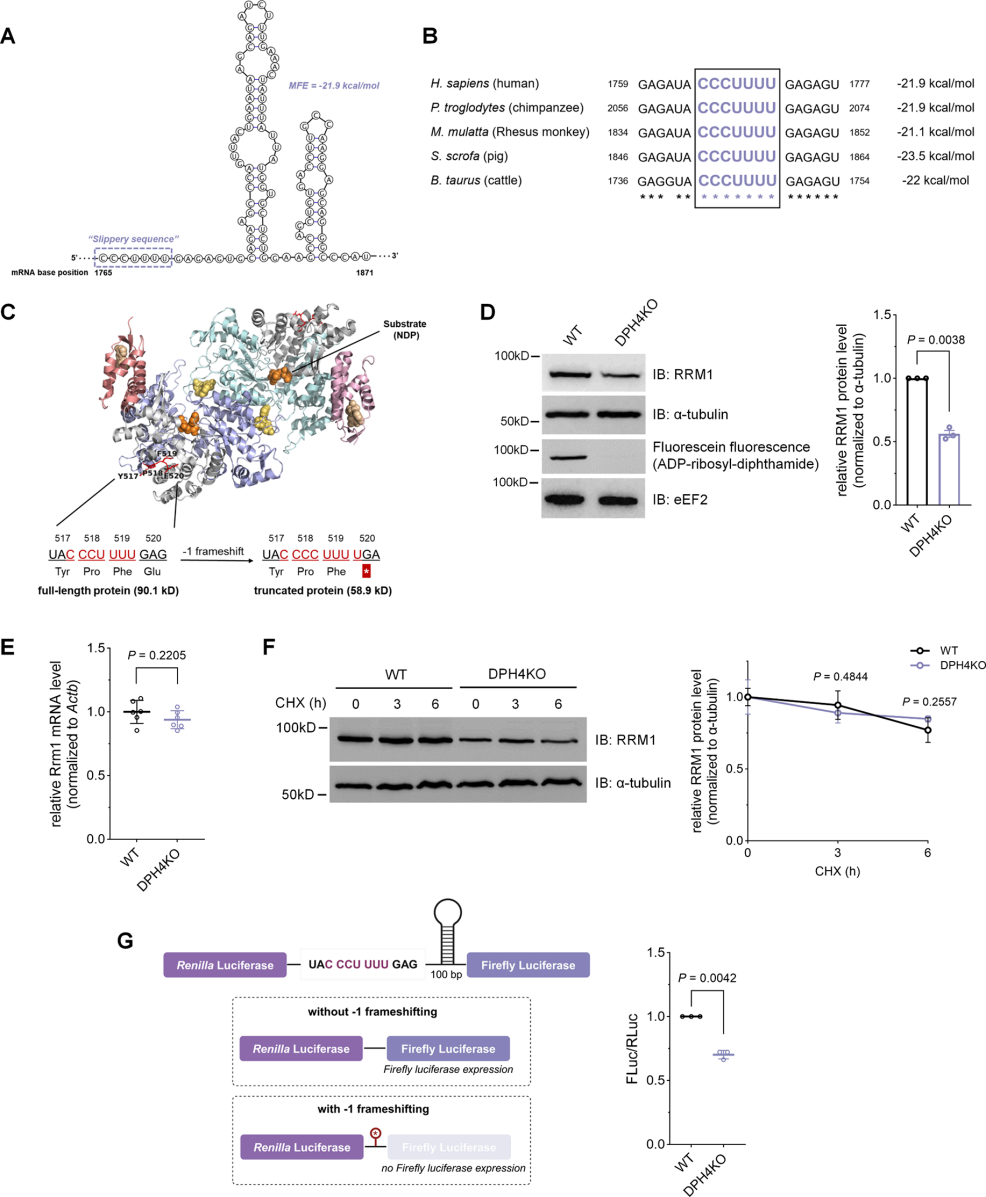
Diphthamide modulates the translation of RRM1 in cells. (A) Predicted −1 frameshifting region of human RRM1 mRNA. mRNA secondary structure and minimum free energy (MFE) were predicted using *ViennaRNA*. (B) Multiple sequence alignment of RRM1 mRNA from five mammalian species. (C) Location of the slippery site (red) mapped to the Cryo-EM structure of the human RRM1 (PDB ID: 6AUI) bound to substrate CDP (orange) and effector dATP (wheat, and a schematic illustration of −1 frameshifting events during RRM1 translation generating an E520* nonsense mutation. The N-terminal cone domain and the conserved core domain are colored light pink/salmon and pale cyan/light blue, respectively; sequences after E520 are colored gray. (D) Immunoblot analysis and quantification of RRM1 protein levels in HEK293T WT and DPH4KO cells. (E) RT-qPCR analysis of RRM1 mRNA levels in HEK293T WT and DPH4KO cells. (F) CHX (100 μg/mL) was added to HEK293T WT and DPH4KO cells and chased for 3 or 6 h. Immunoblot was applied to assess RRM1 degradation rates, and RRM1 protein levels were quantified at each time point. (G) Dual-luciferase reporter assay to confirm the slippery sequence of RRM1. The frameshifting rates were determined by measuring the Firefly to *Renilla* luciferase activity ratios. Representative data from three biologically independent experiments. Data with error bars are mean ± s.d. *P* values are determined using unpaired Welch’s *t* test.

RRM1 encodes a critical regulatory subunit of ribonucleotide reductase, an enzyme that plays a crucial role in the conversion of ribonucleoside diphosphates (NDPs) to deoxyribonucleoside diphosphates (dNDPs), which are converted to deoxyribonucleoside triphosphates, serving as the starting materials for DNA synthesis.^5^ Our computational analysis revealed a potential −1 frameshifting site within the mRNA of human RRM1, characterized by a heptanucleotide slippery sequence “CCCUUUU”, a spacer, and an mRNA secondary structure with an MFE value of −21.9 kcal/mol (Figure 6A). Multiple sequence alignments indicated the conservation of this hypothesized frameshifting site across mammalian species, emphasizing its potential functional relevance (Figure 6B). We hypothesized that loss of diphthamide in eEF2 would promote the −1 frameshifting event at this slippery sequence during RRM1 protein translation, resulting in an E520* nonsense mutation immediately after the slippery sequence and producing a truncated protein (Figure 6C). Subsequently, we mapped the predicted −1 frameshifting site to the cryo-EM structure of the human RRM1 protein. The truncated sequences following the slippery site form part of the substrate (i.e., NDPs) binding pocket of RRM1 (Figure 6C), suggesting that the function of RRM1 is likely inhibited after the −1 frameshifting event.

To further validate the small heavy-to-light abundance ratio (i.e., low RRM1 abundance in DPH4KO cells relative to that in WT) observed in the SILAC experiment, we examined the endogenous RRM1 protein levels in HEK239T WT and DPH4KO cells. Depletion of diphthamide in cells led to a markedly lower RRM1 protein level (Figure 6D). On the other hand, RT-qPCR revealed no significant difference in RRM1 mRNA levels between WT and DPH4KO cells (Figure 6E). Additionally, the cycloheximide (CHX) chase assay demonstrated that diphthamide deficiency did not alter the RRM1 degradation rates (Figure 6F). These findings collectively suggest that the decreased abundance of RRM1 protein is primarily attributed to translational change rather than differential transcription or protein degradation.

We then utilized a dual-luciferase reporter system to verify that the translational suppression of RRM1 in diphthamide-deficient cells is due to −1 frameshifting. We inserted the slippery sequence (CCCUUUU) and the adjacent 100 nucleotide stem-loop region of human RRM1 mRNA between Firefly and *Renilla* luciferases (Figure 6G). Since −1 frameshifting introduces a stop codon immediately after the slippery sequence, leading to premature translation termination, we anticipated a reduction in Firefly luciferase (FLuc) activity in diphthamide-deficient cells. Indeed, the reporter system revealed a significant decrease in the FLuc/RLuc ratio in DPH4KO cells (Figure 6G). Therefore, we concluded that diphthamide promotes RRM1 translation by preventing −1 frameshifting events.

### Diphthamide Affects Cellular DNA Replication Stress through RRM1

**Figure 7:**
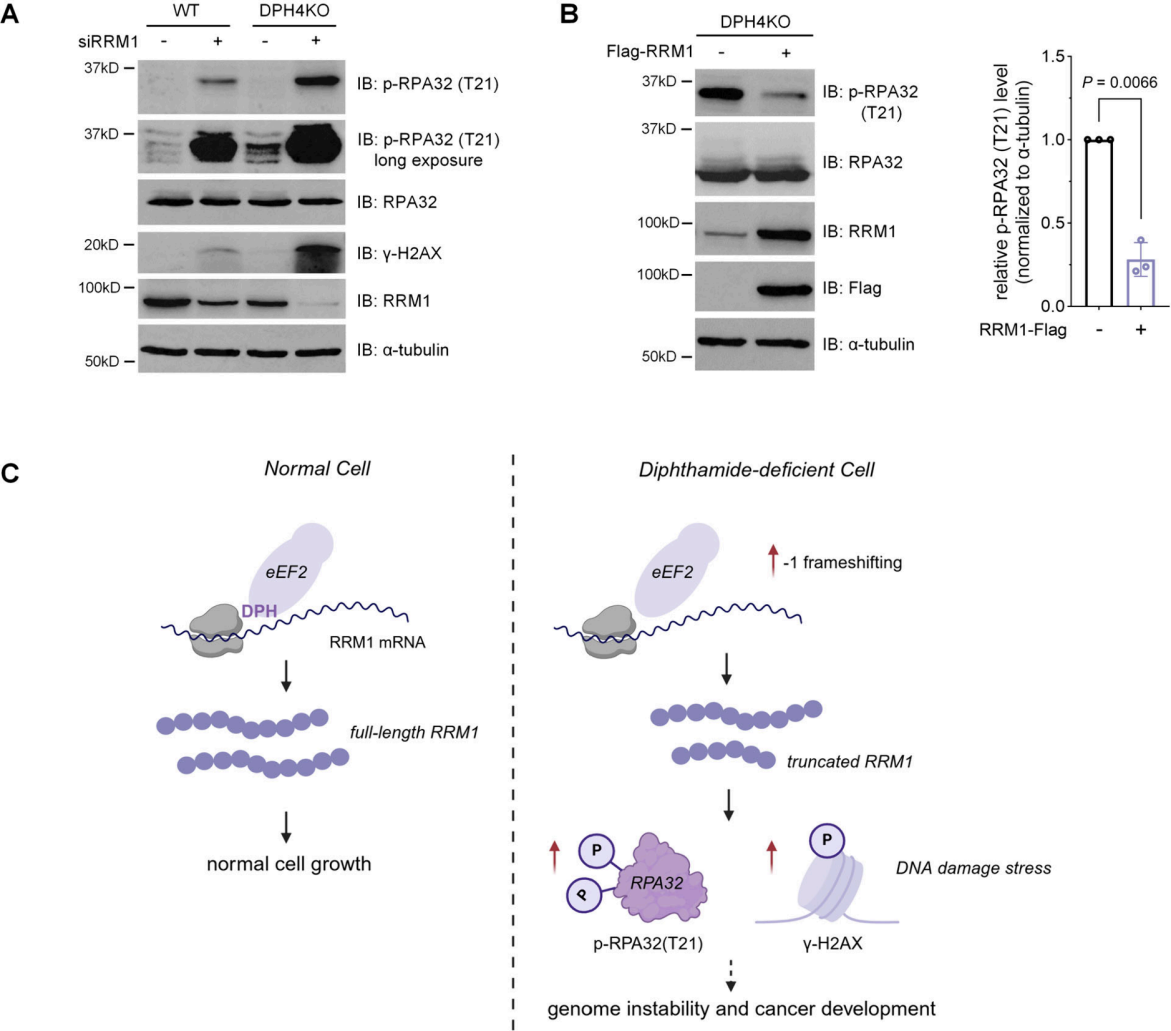
Diphthamide affects cellular DNA replication stress through RRM1. (A) Immunoblot analysis of γ-H2AX and p-RPA32 (Thr21) levels in HEK293T WT and DPH4KO cells with RRM1 siRNA knockdown. (B) Immunoblot analysis and quantification of p-RPA32 (Thr21) levels in HEK293T DPH4KO with RRM1-Flag overexpression. (C) Proposed model for diphthamide’s role in affecting mammalian cell DNA replication stress through modulating RRM1 translation. Data with error bars are mean ± s.d. *P* values are determined by the unpaired Welch’s *t* test.

To investigate whether the observed DNA damage and replication stress in DPH-deficient cells were due to decreased RRM1 protein levels, we conducted a series of experiments involving manipulation of RRM1 expression. First, we transiently knocked down RRM1 using a small interfering RNA (siRNA) in both HEK293T WT and DPH4KO cells. The knockdown of RRM1 resulted in a significant increase in γ-H2AX and pRPA32-Thr21 levels in both cell types (Figure 7A), indicating elevated DNA damage and replication stress. This experiment demonstrated that the reduced RRM1 protein level is sufficient for cellular DNA replication stress.

Next, we explored whether restoring RRM1 levels could mitigate the replication stress observed in DPH-deficient cells. We achieved this by overexpressing RRM1 in DPH4KO-treated HEK293T DPH4KO cells. Notably, rescuing the RRM1 protein level led to a marked decrease in pRPA32-Thr21 levels (Figure 7B), suggesting that the decreased RRM1 level is necessary for the replication stress caused by diphthamide deficiency. Together, these data indicate that the diphthamide deficiency contributes to cellular DNA replication stress by decreasing the RRM1 protein level.

## Discussion

In this study, we developed a novel systematic strategy to identify diphthamide-affected protein targets in human cells by leveraging computational prediction and SILAC quantitative proteomics. Besides, we have developed an approach to directly detect −1 frameshifted peptides generated due to diphthamide deficiency by leveraging an *in-silico* library of frameshifted proteins to search for peptides detected in MS directly. This methodology offers a direct and high-throughput way to detect peptides from frame-shifted proteins; however, it does have certain limitations. Frameshifted proteins may contain motifs prone to misfolding and degradation to maintain proteostasis, resulting in their low abundance and thus making it difficult to detect by MS. Additionally, −1 frameshifting could lead to the immediate generation of a stop codon, resulting in no or very few neopeptides that can be detected in MS. RRM1 is such an example. Nonetheless, these limitations can be addressed in the future. For example, treating cells with proteasomal and lysosomal inhibitors could impede the degradation of frameshifted proteins, aiding in detecting −1 frameshifted peptides. Employing multiple proteases for peptide digestion and implementing deep fractionation for LC-MS/MS analysis could also enhance the coverage of specific proteins, potentially overcoming these challenges.

Predicting and identifying protein targets whose translation depends on diphthamide has been done for yeast proteins.^35^ Our study extends the understanding of the functional importance of diphthamide to humans. Future studies can delve deeper into verifying other identified protein targets of diphthamide. This workflow can also be applied to different cell lines or tissues to uncover diphthamide’s cell- and tissue-specific protein targets, which may provide further insights to understand the disease relevance. Furthermore, using *in-silico* frameshifted libraries in proteomics studies could enhance the identification of other targets in a more direct manner, offering promising avenues for continued investigation.

The diphthamide modification in eEF2 is known to maintain translational fidelity by suppressing −1 frameshifting.^14,15,17^ Our study finds that RRM1, an essential ribonuclease reductase subunit, undergoes a translational decrease in diphthamide-deficient mammalian cells. Notably, this −1 frameshifting signal observed in human RRM1 is not conserved in its yeast homologue, RNR1, which does not show a similar decrease in diphthamide-deficient strains (Figure S5). This suggests that the modulation of RRM1 translation may have specifically evolved in higher mammals (Figure 6B). RRM1 functions as an enzyme that converts ribonucleoside diphosphate to deoxyribonucleoside diphosphate, thereby playing a vital role in maintaining genomic stability. Specifically, RRM1 provides dNTPs for DNA replication during the S-phase and aids in DNA repair under genotoxic stress conditions. Our results indicate that the reduction of the RRM1 protein level during diphthamide deficiency correlates with signs of genomic instability, such as increased γ-H2AX levels in the nucleus and the induction of DNA replication stress as indicated by phosphorylated RPA32. Our study, therefore, connects diphthamide deficiency to DNA damage/replication stress, suggesting a potential mechanism for genome instability. Notably, diphthamide has previously been linked to cancer, with the *DPH1* gene initially identified as a tumor suppressor gene (*OVCA1*); heterozygosity loss of *OVCA1* has been associated with various cancers, particularly ovarian cancer.^24,27,37^ Given that genomic instability resulting from DNA damage and DNA replication stress is associated with cancer progressions,^4,38^ our results provide a potential explanation for how diphthamide deficiency promotes tumors (Figure 7C).

Our results also provide an explanation for why diphthamide deficiency syndrome is characterized by developmental abnormalities such as CNS malformations and dysmorphic features.^16^ The embryonic development process is highly sophisticated and requires precise timing for pluripotency, cellular differentiation, and cell proliferation.^39^ However, diphthamide-deficiency-induced DNA damage, replication stress, and reduced cell proliferation can significantly disrupt these development processes. Therefore, our results align with the observations that mice with diphthamide deficiency show embryonic development defects, which leads to embryonic lethality,^24,25,37^ and that humans with mutations in DPH genes show disorders in development.^26^ Overall, our study elucidates a molecular mechanism underlying the clinical relevance of diphthamide and its implications for disease pathogenesis.

## Materials and Methods

### Reagents, Antibodies, and Plasmids

Silver stain kit (ThermoFisher #24612) was used to detect protein in the denaturing polyacrylamide gel. pCDNA3-RRM1-Flag plasmid was obtained from GenScript (OHu19014); pFA6a-6xGLY-3xFLAG-HIS3MX6 was a gift from Mark Hochstrasser (Addgene plasmid #20753), and pAGH10 - RlucFluc minus 1 HIV-1 was a gift from Isha Jain (Addgene plasmid #198224). siRNAs were obtained from ThermoFisher (siRRM1, s12358) or Santa Cruz Biotechnology (control siRNA-A, sc-37007). Flag-horse radish peroxidase (Sigma no. A8592) was used to immunoblot for Flag-conjugated proteins at 1:5000 dilution. Primary antibodies from Cell Signaling Technology: RRM1(#8637), RPA32 (#35869), HA (#3724), eEF2 (#2332), γH2AX (#9718), GAPDH (#2118), α-tubulin (#3873); Abcam: p-RPA32 T21 (#ab61065); Proteintech: RABGEF1 (#12735-1-AP); Santa Cruz: β-actin (sc-47778); and Novus Biological: DPH4 (#NBP1–87969) were used for immunoblotting at 1:1000 dilution.

### Computational Profiling of Human Transcript Genome Database

The human consensus coding sequence (CCDS) database was obtained from NCBI CCDS database Web site (https://www.ncbi.nlm.nih.gov/projects/CCDS/) (release 24). A slippery sequence with X XXY YYZ was searched, with X denoting any nucleotide, Y denoting A or U, and Z denoting A, U, or C. The folding minimum free energy of 100 bp after the slippery sequence was calculated using *Vienna RNA*.^33^ Each gene with at least one slippery sequence was identified as a potential −1 frameshifting site if the minimum MFE of the slippery sequence was less than −10 kcal/mol. Computational analysis was performed using R and the Biostrings package.

### Generation of *in-Silico* Frameshifted Libraries

A list of CCDS transcripts containing slippery sequences with minMFE less than −10 kcal/mol was used to generate the library. The *in-silico* −1 frameshifting event was achieved by adding an additional first base pair RNA to the start of the slippery sequence on mRNA. For transcripts with multiple slippery sequence motifs, each slippery sequence motif was considered individually (i.e., *in-silico* −1 frameshifting was performed at each site), and multiple sequences of the resulting frameshifted protein sequences were added to the library sequentially. The library of translated proteins was then output in a fast format as “Frameshifted_Library”. To facilitate the data analysis, frameshifted protein sequences after the slippery sequence motif of each transcript were also output as “Frameshifted_Library_afterslipseq”. The two libraries were then used as search files for peptide identification (see Mass Spectrometry Data Processing section for details).

### Generation and Culture of HEK293T DPH4KO Cells

The HEK293T DPH4KO cell line was generated in accordance with a published protocol.^40^ HEK293T DPH4KO cells were cultured in Dulbecco’s modified Eagle medium (DMEM) (Thermo) supplemented with 10% (v/v) heat-inactivated calf serum (Sigma #C8056).

### NAD^+^ Analog Labeling of EF2 to Detect Levels of Diphthamide Modification

The diphtheria toxin-mediated ADP-ribosylation assay to detect the diphthamide modification level was performed with fluorescein-NAD^+^ (biotechne/Fisher #6574) and purified diphtheria toxin as previously described.^41^

### Stable Isotope Labeling by Amino Acids (SILAC)

HEK293T wild-type or DPH4KO was cultured in DMEM for SILAC (Thermo #88364) supplemented with dialyzed fetal bovine serum (Thermo #26400044). For light-isotope-labeled cells, l-arginine (Sigma #A8094) and l-lysine (Sigma #L9037) were added to the media. For heavy-isotope-labeled cells, l-arginine-^13^C_6_,^15^N_4_ hydrochloride (Sigma #608033) and l-lysine-^13^C_6_,^15^N_2_ hydrochloride (Sigma #608041) were added to the media. SILAC samples were prepared in triplicates. Equal numbers of light-labeled and heavy-labeled cells were mixed and lysed in a RIPA lysis buffer. The lysates were subjected to reduction, alkylation, and trypsin digestion using an S-Trap proteomics sample preparation kit (Protifi). The resulting peptides were subjected to LC-MS/MS analysis.

### Liquid Chromatography and Mass Spectrometry

The tryptic digests of the SILAC samples were reconstituted in 2% acetonitrile with 0.5% formic acid (FA) for nanoLC-ESI-MS/MS analysis. The analysis was carried out using an Orbitrap Fusion Tribrid (Thermo-Fisher Scientific, San Jose, CA, USA) mass spectrometer equipped with a nanospray Flex Ion Source and coupled with a Dionex UltiMate 3000 RSLCnano system (Thermo, Sunnyvale, CA, USA). Peptide samples (10 μL) were injected onto a PepMap C-18 RP nano trapping column (5 μm, 100 μm i.d. × 20 mm) with nanoViper fittings at a 20 μL/min flow rate for rapid sample loading and then separated on a PepMap C-18 RP nanocolumn (2 μm, 75 μm i.d. × 25 cm) at 35 °C. The tryptic peptides were eluted in a 90 min gradient of 5% to 35% ACN in 0.1% formic acid at 300 nL/min, followed by a 7 min ramping to 90% ACN–0.1% FA and an 8 min hold at 90% ACN–0.1% FA. The column was re-equilibrated with 0.1% FA for 25 min prior to the next run. The Orbitrap Fusion was operated in positive ion mode with spray voltage set at 1.6 kV and source temperature at 275 °C. External calibration for FT, IT, and quadrupole mass analyzers was performed. In data-dependent acquisition (DDA) analysis, the instrument was operated using an FT mass analyzer in MS scan mode to select precursor ions followed by 3 s “top speed” data-dependent CID ion trap MS/MS scans at 1.6 *m*/*z* quadrupole isolation for precursor peptides with multiple charged ions above a threshold ion count of 10,000 and normalized collision energy of 30%. MS survey scans were done at a resolving power of 120,000 (fwhm at *m*/*z* 200), for the mass range of *m*/*z* 375–1600. Dynamic exclusion parameters were set at 35 s of exclusion duration with ±10 ppm exclusion mass width. All data were acquired under the Xcalibur 4.4 operation software (Thermo-Fisher Scientific).

### Mass Spectrometry Data Processing

The DDA raw files with MS and MS/MS spectra were subjected to database searches using Proteome Discoverer (PD) software (version 3.0.0.757, Thermo Fisher Scientific, Bremen, Germany). A Minora feature detector was used for precursor ion-based quantification. The database search was conducted against three databases, including the *Homo sapiens* Uniprot database, which contains 26,019 sequences, “Frameshifted_Library”, which contains 25,677 sequences, and “Frameshfted_Library_afterslipseq”, which contains 14,692 sequences. The peptide precursor tolerance was set to 10 ppm, and fragment ion tolerance was set to 0.6 Da. Two Sequest HT nodes were applied. In the SILAC light node, oxidation (M), Acetyl (N-terminus), Met-loss (M), and Met loss+Acetyl (M) were set as variable modifications, whereas carbamidomethyl (C) was set as fixed modification. In the SILAC heavy node, oxidation (M), Acetyl (N-terminus), Met-loss (M), and Met loss+Acetyl (M) were set as variable modifications, whereas R10 (+10.008 Da), K8 (+8.014 Da), and carbamidomethyl (C) were set as fixed modifications. Only high-confidence peptides defined by Sequest HT with a 1% FDR by Percolator were considered for confident peptide identification. Relative quantitation of identified proteins of heavy versus light labeled was determined by the Precursor Ions Quantifier node for SILAC 2plex (Arg10, Lys8) within the Precursor Quan workflow in PD 3.0. The precursor abundance intensity for each peptide identified by MS/MS in each replicate was automatically determined, and their unique plus razor peptides for each protein in each replicate were used to calculate the heavy/light ratios. “Nested design” was applied for quantification, the raw data of “abundances by bio. Rep” was extracted, and the mean of the triplicated samples was used for heavy/light ratio calculation. The final protein group list was further filtered with 5 ppm for identified peptides and two peptides per protein in which only #1-ranked peptides within top scored proteins were used. For statistical analysis of SILAC data, log2 transformation of the heavy/light ratio was applied in the calculation to represent the fold change in the data set. A Student’s *t* test was performed on the data to compare experimental groups, and the significant cutoff was set to a *P*-value of less than 0.05.

### Immunofluorescence Staining

Cells were seeded onto a poly-d-lysine-coated 35 mm dish with a 14 mm glass diameter coverslip (Mattek #P35GC-1.5-14-C) on the bottom and incubated overnight. The cells were washed, fixed with 4% paraformaldehyde/PBS, and permeabilized with 0.2% Triton X-100/PBS. Permeabilized cells were blocked with 5% BSA/0.1% Triton X-100/PBS and then incubated overnight with primary antibody for γ-H2AX (CST #9718) or p-RPA32 T21 (Abcam #ab61065) at 1:100 dilution. Secondary antibodies conjugated with Alexa Fluor 488 (Thermo #A-11034) were used at 1:1000 dilution. Hoechst 33342 (Invitrogen #3570) was used for nuclei staining. Images were obtained using an LSM880 confocal microscope (Carl Zeiss, Inc.) and processed with ImageJ.

### Quantitative Reverse Transcription PCR (RT-qPCR)

Total RNA of HEK293T WT and DPH4KO cells were extracted with the E.Z.N.A. total RNA kit I (Omega BioTek #R6834-00S) according to the manufacturer’s instruction. cDNA was synthesized with a SuperScript VILO cDNA synthesis kit (Invitrogen #11754050). Quantitative PCR was performed using 2x Universal SYBR Green Fast qPCR mix (Abclonal no. RK21203) by QuantStudio 7 Flex real-time PCR system. Primers used for RT-qPCR are listed in Table S1.

### Cyclohexamide Chase Assay

HEK293T cells were plated in a six-well plate 24 h prior to the treatment, the old medium was discarded, and then the cells were treated with fresh medium containing cycloheximide (Sigma) at a 100 μg/ml concentration. Cells were then harvested at the indicated time points (0, 3, and 6 h), followed by lysis with RIPA buffer and immunoblot analysis.

### –1 Frameshifting Dual-Luciferase Reporter Assay

The dual-luciferase reporter plasmid for the RRM1 −1 frameshifting region was generated via site-directed mutagenesis from pAGH10 - RlucFluc minus 1 HIV-1 (Addgene #198224) with the following primers: 5′-actatttattatggtgctctggaagccagctgtgaccttgccaaggagcagggcccatacATGGAAGACGCAAAAAAC-3′ (forward) and 5′-ttcaaagatctgcttattcagtaactgggcttctgcactctcaaaagggtatctcatcagTTGCTCATTCTTCAGAAC-3′ (reverse). HEK293T cells were transfected with the above plasmid and harvested 24 h post-transfection. The Firefly and *Renilla* luciferase luminescence was measured using the Dual-Glo luciferase assay (Promega E2920) according to the manufacturer’s instructions.

### Transfection of Plasmids and siRNAs

HEK293T cells were plated in a six-well plate 24 h prior to the transfection. Plasmids were transfected using polyethylenimine hydrochloride (PEI, Polysciences) at a ratio of 1:3 for DNA:PEI. siRNAs were transfected by Lipofectamine RNAiMAX (Invitrogen) following the manufacturer’s instruction. Cells were harvested 48 h post-transfection, and the cell lysates were subjected to immunoblot analysis.

### Cell Growth Curve and Doubling Time

A total of 100,000 HEK293T and DPH4KO cells were plated in 10 cm cell culture dishes and incubated overnight. Cells were then detached from plates using Trypsin-EDTA (Thermo #15090046), and the total number of cells were counted using an automated cell counter (BioRad #TC20) at indicated time points. Doubling time was calculated using the equation , where *t* is total growth time, *N*_0_ is initial number of cells, and *N*_*t*_ is the final number of cells at time *t*.

### Knock-in of Endogenous Yeast Proteins with 3xFlag Tag and Yeast Protein Extraction

The strains expressing endogenous 3xFlag-tagged RNR1 were generated using homologous recombination as previously described^42^ with the following primers: 5′-TGCTATTGATAACCCAGAAGCTTGTGAAATGTGTTCGGGTcggatccccgggttaattaa-3′ (forward) and 5′-TTTGATAAATAGAATTGAAGAAATAAAAACTTAGCCCTCAgaattcgagctcgtttaaac-3′ (reverse). Yeast BY4741 WT or diphthamide-deficient strains were transformed with the amplified PCR product and plated on synthetic complete agar plates with a histidine dropout for selection. Yeast total protein was extracted as previously described using NaOH–trichloroacetic acid precipitation.^35,43^

### Statistics

GraphPad Prism software was used for statistical data analysis.

## Data Availability

The proteomic raw data are available via ProteomeXchange with the identifier PXD051718.

## Supporting Information Available

SILAC proteomics and computational prediction results (XLSX)
Transcripts with slippery sequences (XLSX)
Supplementary Figures S1–S5, uncropped blots for all immunoblotting experiments, and RT-qPCR primer sequences (PDF)
Transparent Peer Review report available (PDF)
