# Supplementary material for: Loss of Diphthamide Increases DNA Replication Stress in Mammalian Cells by Modulating the Translation of RRM1
Source: ACS Cent Sci. 2024 Sep 6;10(10):1835–47. doi: 10.1021/acscentsci.4c00967 (PMC11503486; doi:10.1021/acscentsci.4c00967)
Supplement: Supplementary file 3 — oc4c00967_si_003.pdf [file oc4c00967_si_003.pdf]

Supporting Information for

Loss of diphthamide increases DNA replication stress in mammalian cells by modulating the translation of RRM1

Jiaqi Zhao[a,1], Byunghyun Ahn[a,b,1], and Hening Lin[a,b,c,*]

[a] Department of Chemistry and Chemical Biology, Cornell University, Ithaca, NY 14853, United States

[b] Department of Molecular Biology and Genetics, Cornell University, Ithaca, NY 14853, United States

[c] Howard Hughes Medical Institute, Cornell University, Ithaca, NY 14853, United States

[1] These authors contributed equally to this work

* Corresponding author: Hening Lin

Email: hl379@cornell.edu

This file includes:

Supplementary Figures S1-S5

Uncropped blots for all immunoblotting experiments Figure S6-S11

Supplementary Table S1-S2

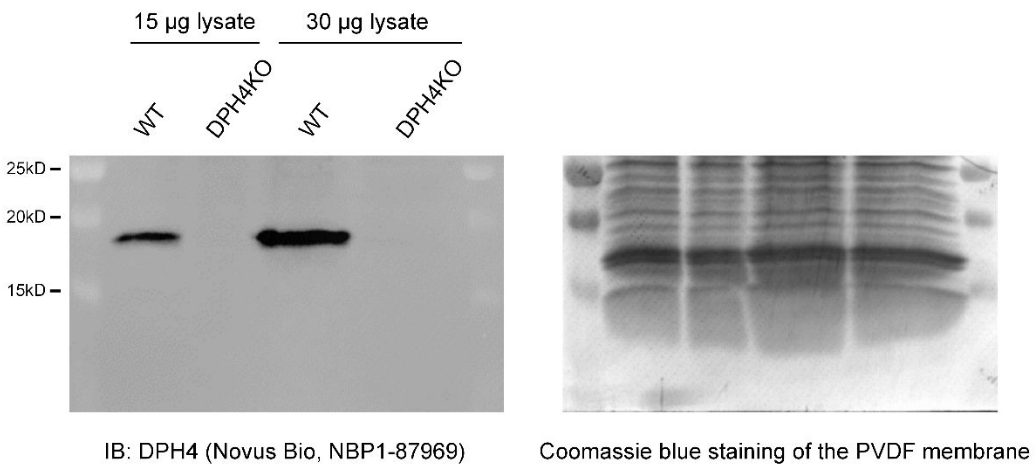

Figure S1. Verification of the HEK293T-DPH4 CRISPR KO cell line. HEK293T-WT and DPH4KO cell lysates were resolved by SDS-PAGE, and endogenous DPH4 protein levels were assessed by immunoblotting. The loading of each sample was assessed by the Coomassie blue staining of the PVDF membrane.

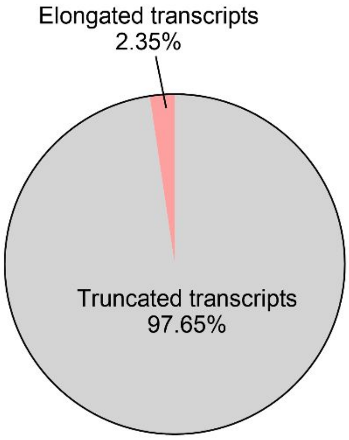

Figure S2. Ratio of the truncated and elongated transcripts after the hypothetical -1 frameshifting event. A truncated transcript indicates a stop codon is generated prior to the one in the original reading frame, while an elongated transcript indicates a stop codon is generated after the one in the original reading frame.

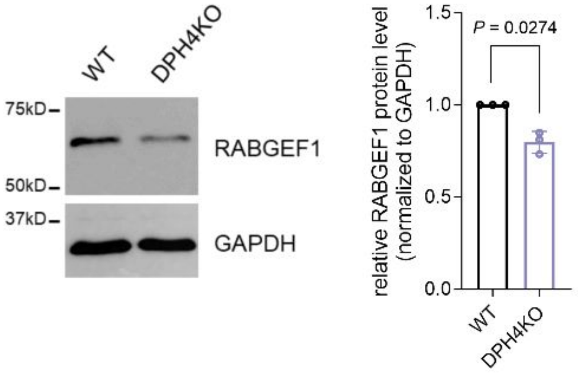

Figure S3. Immunoblot analysis and quantification of RABGEF1 protein levels in HEK293T WT and DPH4KO cells. Data with error bars are mean ± s.d. *P* values are determined by the unpaired Welch's t-test.

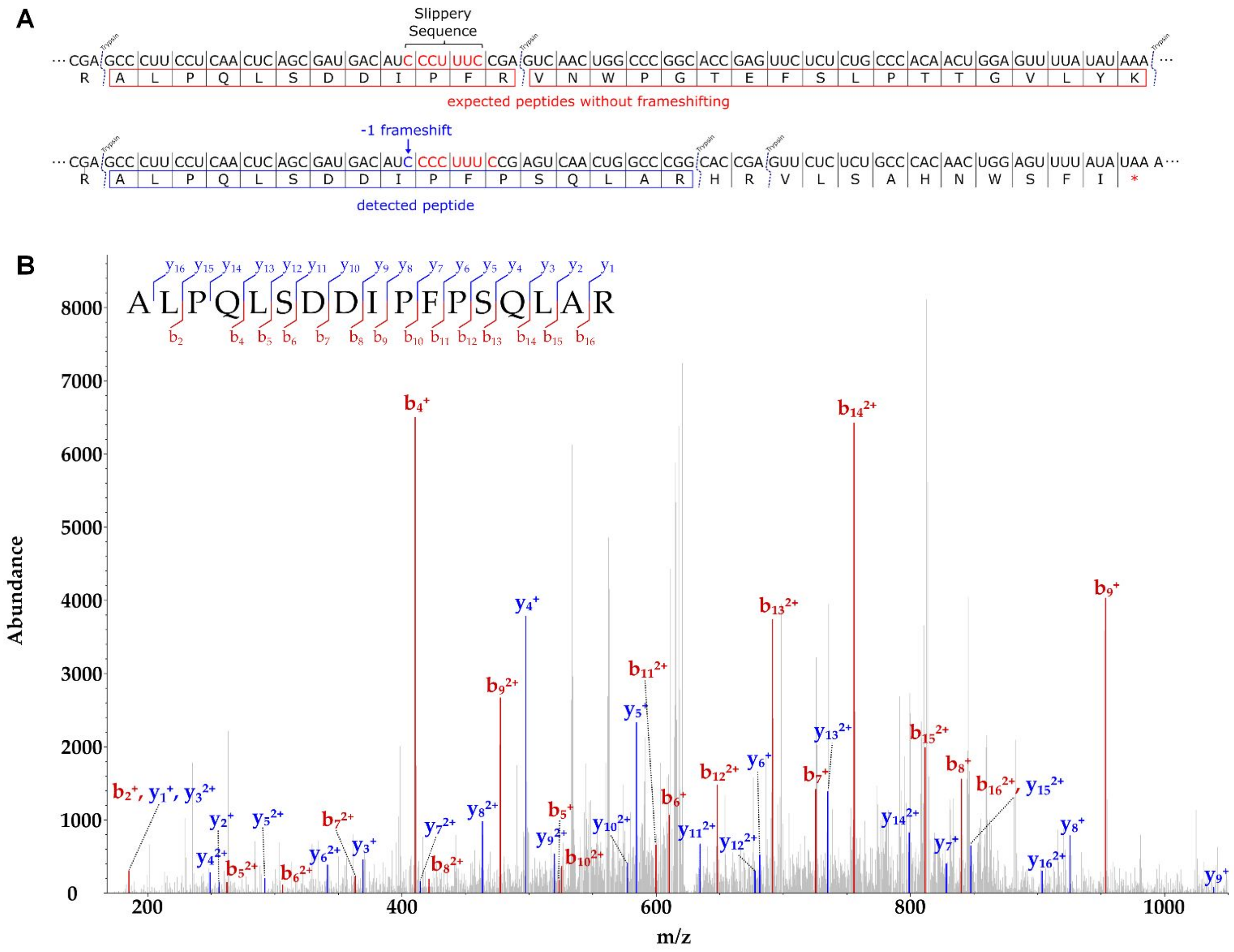

Figure S4. Identification of -1 frameshifted peptides from ERLEC1 protein in HEK293 DPH4KO cells. (A) Sequence alignment of ERLEC1 mRNA reading frame without or with predicted -1 frameshifting event, ALPQLSDDIPFPSQLAR peptide and a nonsense mutation are generated after -1 frameshifting. The slippery sequence is labeled in red. Theoretical trypsin-digested peptides from the original reading frame are highlighted in red boxes, and the detected -1 frameshifted peptide is highlighted in the blue box. **(B)** MS/MS spectrum for the frameshifted peptide ALPQLSDDIPFPSQLAR detected in the HEK293T DPH4KO sample.

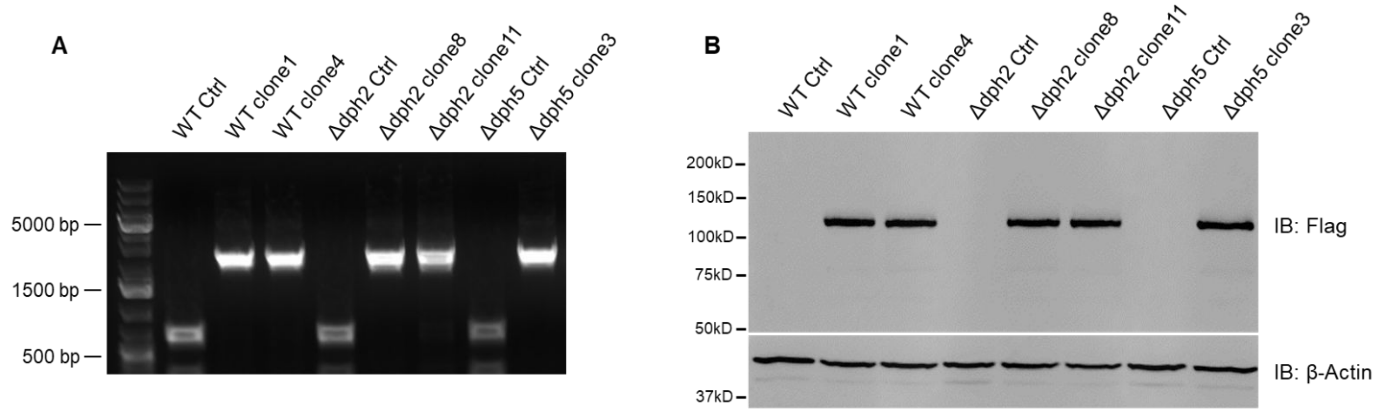

Figure S5. RNR1 levels in normal and diphthamide-deficient yeast strains. (A) Endogenous RNR1 in yeast BY4741 WT, Δdph2, and Δdph5 strains was tagged with a C-terminal 3XFlag tag via genome editing. Primers from 500 bp before (forward) and 100 bp after (reverse) the RNR1 stop codon were used to amplify yeast genomic DNA. A shift from ~600bp to ~2200bp (addition of 3xFlag and the HIS3MX6 selection marker) was observed in the knocked-in strains. The PCR products were purified and further confirmed through sequencing. **(B)** Yeast cells were cultured in YPD broth to OD_{600} = 0.6. Total protein was extracted via NaOH-trichloroacetic acid precipitation. Endogenous RNR1 protein levels were assessed through immunoblotting. Representative data of two biological replicates.

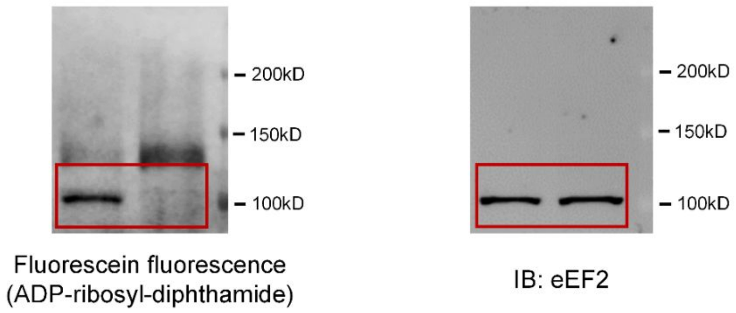

Figure S6. Complete blots for Figure 1B.

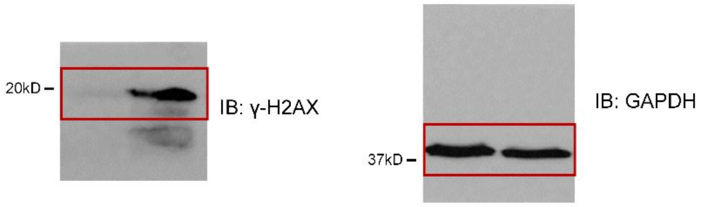

Figure S7. Complete blots for Figure 2A.

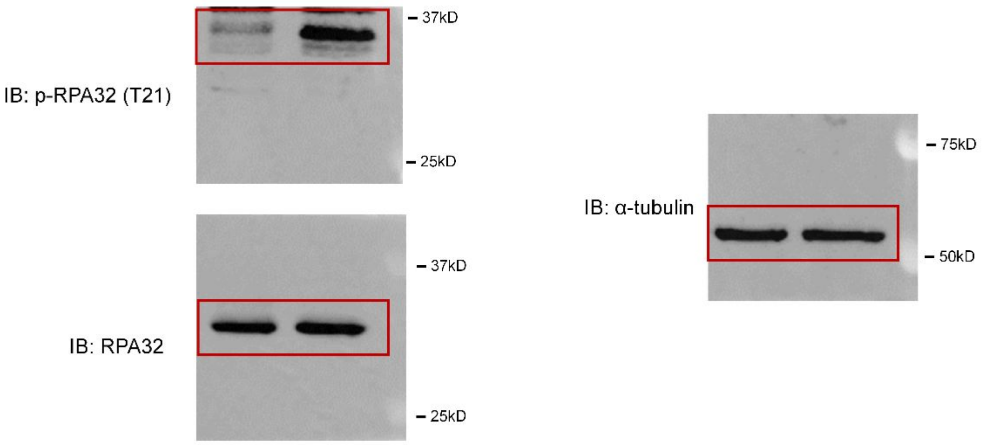

Figure S8. Complete blots for Figure 2C.

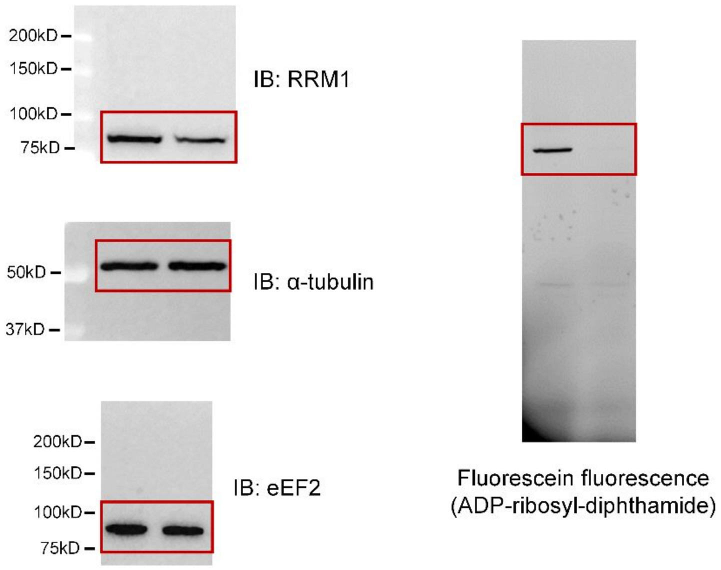

Figure S9. Complete blots for Figure 6D.

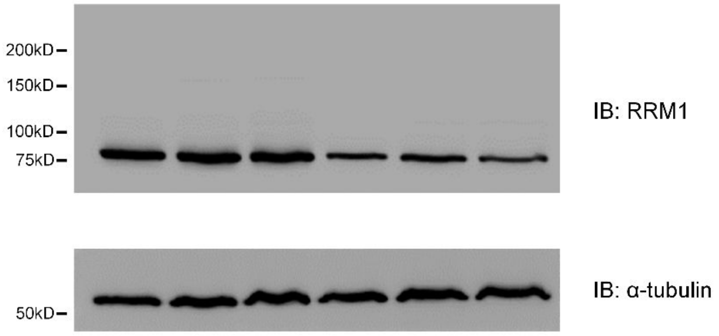

Figure S10. Complete blots for Figure 6F.

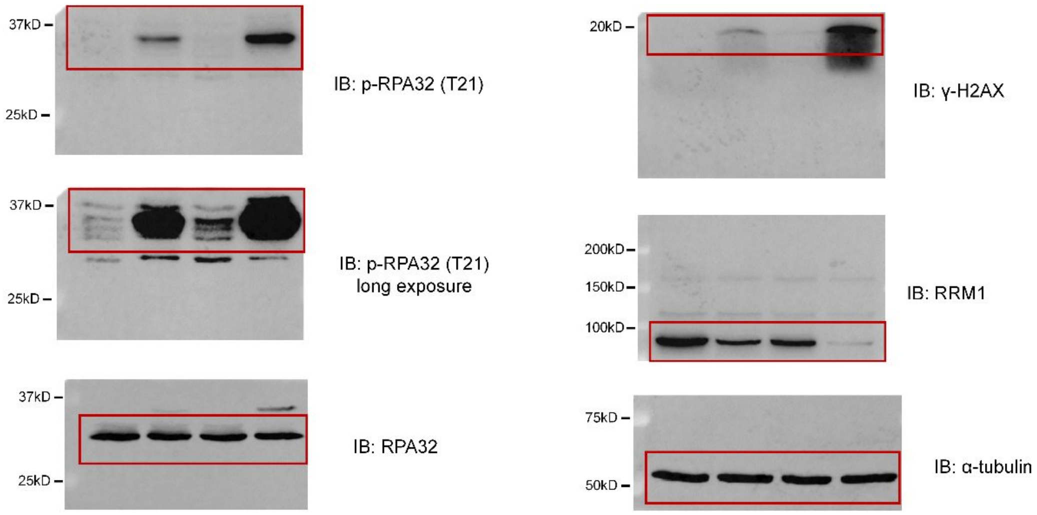

Figure S11. Complete blots for Figure 7A.

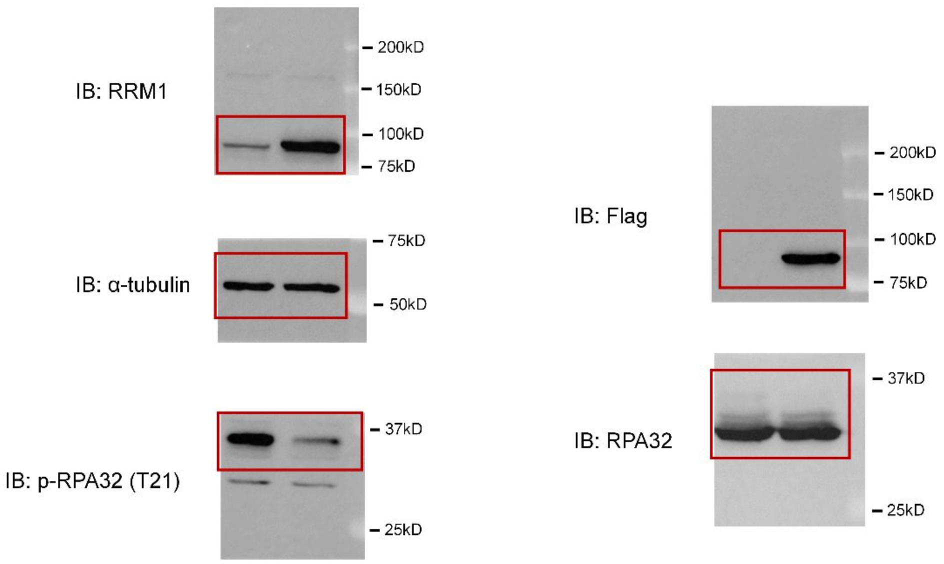

Figure S12. Complete blots for Figure 7B.

Table S1. Examples of SILAC-identified proteins with increased levels in DPH4KO cells.

Protein name	Uniprot ID	Protein mw (kDa)	SILAC H/L ratio
RPL22	P35268	14.8	2.46
UBE2V1	Q13404	16.5	2.56
TRMT112	Q9UI30	14.2	100
PFDN4	Q9NQP4	15.3	100
CYB5A	P00167	15.3	100
MRPL41	Q8IXM3	15.4	100
SCAMP3	O14828	38.3	100
DHRS7	Q9Y394	38.3	100
QNG1	Q5T6V5	39	100

Table S2. Sequences of RT-qPCR primers

Genes	Primers	Sequences (5' to 3')
Human *RRM1*	Forward	AAAGGAAGAGCAGCGTGCCAGA
	Reverse	CCTCATCCAGACCAGGACACT
Human *ACTB*	Forward	CACCATTGGCAATGAGCGGTTC
	Reverse	AGGTCTTTGCGGATGTCCACGT
