# Supplementary material for: Loss of Diphthamide Increases DNA Replication Stress in Mammalian Cells by Modulating the Translation of RRM1
Source: ACS Cent Sci. 2024 Sep 6;10(10):1835–47. doi: 10.1021/acscentsci.4c00967 (PMC11503486; doi:10.1021/acscentsci.4c00967)
Supplement: Supplementary file 4 — oc4c00967_si_004.pdf [file oc4c00967_si_004.pdf]

oc-2024-00967k.R1

Name: Peer Review Information for "Loss of diphthamide increases DNA replication stress in mammalian cells by modulating the translation of RRM1"

First Round of Reviewer Comments

Reviewer: 1

Comments to the Author
The manuscript 'Diphthamide regulates mammalian DNA replication stress by modulating the translation of RRM1' by Jiaqi Zhao and colleagues analyzes the influence of diphthamide on translation and potential translational slippage by combining bioinformatics, quantitative proteomics and experimental wet-lab analyses. They identified candidate proteins whose translation appears to be frameshifted in diphthamide-deficient DPH4ko cells. Addressing one of them in more detail, they provide several lines of evidence that -1 frameshift causes RRM1 truncation which leads to replication stress and reduced growth in mammalian HEK293T-DPH4ko cells. The described integrated approach addresses the relevant question if there are 'special' proteins that respond to or sense presence or absence of diphthamide, and with which consequences.
The authors have addressed many parameters that underline their conclusions concerning RRM1 in a detailed manner. However, there are some topics that are unclear or are not described in a comprehensive manner and should be addressed:

1. SILAC experiments: I did not find any note or mentioning of SILAC results for DPH4 (or DNAJC24) which has been inactivated to generate diphthamide deficiency.
--> Please provide the protein ratios for DPH4. DPH4 is also a desired control as it is present in parent cells and must be absent in CRISPR cells.

2. Fig 5A shows not only reduced protein bands in DPHko cells, but also at least 2 bands (37kD, 15kD) that are strongly increased in DPHko.
--> Those should be mentioned & called as candidates if they correspond to DPHko-increased SILAC results. Or explain why such proteins do not appear in SILAC expts.

3. Fig 5E shows that the significance (p-value) of RRM1 is not among the top values that were observed.
--> What about candidates with superior values, were they also addressed and then negative in follow-up experiments? Or were they disregarded as irrelevant because of the replication stress hypothesis – please explain.

4. Fig 6D shows only a small part of the blot with approx. 50% signal reduction for RRM1 protein. A frameshift generated smaller RRM1-E520stop fragment should also be detectable on the blot.
--> Please provide complete blots (suppl.data for all Westerns) and explain in case the truncated RRM1 protein is not detected.

5. Standard to assess -1 frameshift regions are dual reporter assays in the bdifferent reading frames.
--> Such analyses should be performed for the RRM1 region (incl. suggested stemloop/knot) to support the frameshift conclusions and to give some indication as for the level of frameshift.

6. It was previously shown that diphthamide-deficient cells carry elevated ROS levels. Is it possible that ROS is the initial trigger of replication stress and RRM1 follows thereafter?
--> please explain

7. RRM1 is highly conserved from yeast to human.
--> Because the majority of 'biology-related' diphthamide analyses are/were done in yeast, please state if the proposed RRM1 frameshift feature also exist in yeast.

8. Title: "Diphthamide regulates mammalian DNA replication stress by modulating the translation of RRM1"
--> The data do not support physiological 'regulation' I suggest to stick to the data like Loss of diphthamide affects the translation of RRM1 and causes DNA replication stress in HEK293T cells.

9. Introduction: Despite its chemically challenging biosynthetic pathway, diphthamide is exclusively found on one protein eEF2, and evolutionarily conserved in all eukaryotes.
--> Narrowe, et al 2018 provide evidence for absence of diphthamide in eukaryotic parabasilids. Please correct 'all eukaryotes'.

10. Abstract: Employing a novel strategy, we identified diphthamide-regulating protein candidates by combing computational profiling of the human transcriptome and quantitative proteomics.
--> I guess they mean diphthamide regulated candidates as there is no evidence that those proteins regulate the synthesis of diphthamide. And (see comment to title) 'regulation' may not be appropriate (better diphthamide- dependent/modulated/affected throughout the manuscript). 'Combing': please check for and correct typos throughout the manuscript.

11. Abstract & Discussion: 'Our results provide explanations for the association between diphthamide deficiency and cancer'; …. our results provide a logical explanation for why diphthamide deficiency promotes tumors…'
--> Because several other proteins are modulated by presence/absence of diphthamide, incl. some proteins that appear to be strongly increased in DPHko cells (37kD & 15kD see Fig. 5A), I suggest to tone down conclusions that are related to RRM1 and cancer.

Author's Response to Peer Review Comments:

We would like to thank the editor and reviewer for the helpful comments. We have addressed all the comments as detailed below. Editor and Reviewer's comments are shown in black fonts, while our responses are shown in blue fonts.

SYNOPSIS MISSING: The synopsis should be no more than 200 characters (including spaces) and should reasonably correlate with the TOC graphic. The synopsis is intended to explain the importance of the article to a broader readership across the sciences. Please place your synopsis in the manuscript file after the TOC graphic, and label it as "Synopsis."

We have included the synopsis in the manuscript file after the TOC graphic. It reads as the following: "Integration of computational and proteomic methods reveals that the loss of diphthamide leads to DNA replication stress in mammalian cells by modulating RRM1 translation."

TOC MISSING: Provide a TOC image per journal guidelines (3.25 in. × 1.75 in. (8.25 cm × 4.45 cm); on the last page of the Manuscript) with the heading "TOC Graphic" above the graphic. Make sure to designate the file as "Graphic for Manuscript."

We have included the TOC image at the end of the manuscript file.

SI PARAGRAPH: If the manuscript is accompanied by any supporting information for publication, a brief description of the supplementary material is required in the manuscript. The appropriate format is: Supporting Information. Brief statement in non-sentence format listing the contents of the material supplied as Supporting Information.

We have included the SI paragraph before the Acknowledgements section.

SI FILE: Please provide as a separate file, designated SI for Publication. Do not include your SI in the MS file.

We have removed the SI from the manuscript file and prepared separate SI files.

Reviewer: 1

Recommendation: Reconsider after major revisions noted.

Comments:

The manuscript 'Diphthamide regulates mammalian DNA replication stress by modulating the translation of RRM1' by Jiaqi Zhao and colleagues analyzes the influence of diphthamide on translation and potential translational slippage by combining bioinformatics, quantitative proteomics and experimental wet-lab analyses. They identified candidate proteins whose translation appears to be frameshifted in diphthamide-deficient DPH4ko cells. Addressing one of them in more detail, they provide several lines of evidence that -1 frameshift causes RRM1 truncation which leads to replication stress and reduced growth in mammalian HEK293T-DPH4ko cells. The described integrated approach addresses the relevant question if there are 'special' proteins that respond to or sense presence or absence of diphthamide, and with which consequences.

The authors have addressed many parameters that underline their conclusions concerning RRM1 in a detailed manner. However, there are some topics that are unclear or are not described in a comprehensive manner and should be addressed:

1. SILAC experiments: I did not find any note or mentioning of SILAC results for DPH4 (or DNAJC24) which has been inactivated to generate diphthamide deficiency.

--> Please provide the protein ratios for DPH4. DPH4 is also a desired control as it is present in parent cells and must be absent in CRISPR cells.

The reviewer's observation regarding the absence of DPH4 in our SILAC results is correct. DPH4 was not detected in WT or DPH4KO SILAC samples among the approximately 1700 identified proteins. We attribute this to the low abundance of DPH4 in HEK293T cells. To ensure that DPH4 is indeed absent in CRISPR cells, we employed immunoblotting as an alternative method to demonstrate the complete knockout of DPH4 in our CRISPR cells (**Figure R1**). This result supports our conclusion despite the limitations of our SILAC experiment in detecting low-abundance proteins like DPH4. We hope this clarification adequately addresses the reviewer's concern.

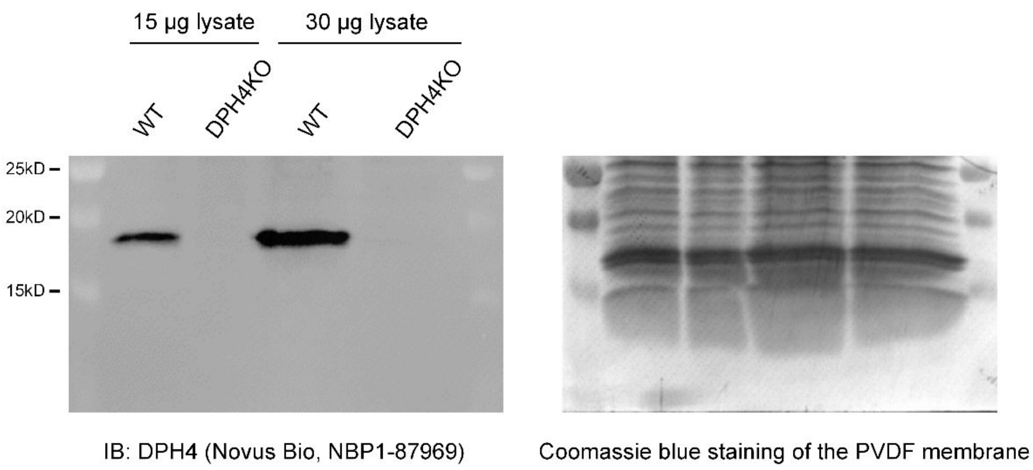

Figure R1. Immunoblotting verification of the HEK293T DPH4 KO cell line. HEK293T DPH4 WT and KO cell lysates were resolved by SDS-PAGE, and endogenous DPH4 protein levels were assessed by immunoblotting. The loading of each sample was assessed by the Coomassie blue staining of the PVDF membrane. This figure is added to the revised supporting materials as **Figure S1**.

2. Fig 5A shows not only reduced protein bands in DPHko cells, but also at least 2 bands (37kD, 15kD) that are strongly increased in DPHko.
--> Those should be mentioned & called as candidates if they correspond to DPHko-increased SILAC results. Or explain why such proteins do not appear in SILAC expts.

We acknowledge the reviewer's observation regarding the proteins with increased levels shown in **Figure 5A**. These proteins were also identified in the SILAC experiment and should indeed be considered as "diphthamide-affected protein candidates". Specifically, proteins approximately 37 kDa and 15 kDa in size, such as TRMT112, PFDN4, SCAMP3, DHRS7, etc., were identified (**Table R1**), including proteins exclusively detected in heavy samples (i.e., DPH4KO cells). We have revised the manuscript to include these proteins in the main text. However, our primary interest in this study lies in the down-regulated proteins based on the "slippery sequence" and -1 frameshifting hypothesis. We believe the up-regulated proteins could be of significant interest for future endeavors.

Table R1. Examples of SILAC-identified proteins with increased levels in DPH4KO cells.

Protein name	Uniprot ID	Protein mw (kDa)	SILAC H/L ratio
RPL22	P35268	14.8	2.46
UBE2V1	Q13404	16.5	2.56
TRMT112	Q9UI30	14.2	100
PFDN4	Q9NQP4	15.3	100
CYB5A	P00167	15.3	100
MRPL41	Q8IXM3	15.4	100
SCAMP3	O14828	38.3	100
DHRS7	Q9Y394	38.3	100
QNG1	Q5T6V5	39	100

*This table is added to the supporting materials as **Table S1**.*

3. Fig 5E shows that the significance (p-value) of RRM1 is not among the top values that were observed.
--> What about candidates with superior values, were they also addressed and then negative in follow-up experiments? Or were they disregarded as irrelevant because of the replication stress hypothesis – please explain.

We thank the reviewer for the insightful comment. We understand the importance of addressing the relevance of candidates with superior values in **Figure 5E**.

When we initially analyzed the SILAC proteomics data, most candidates were disregarded as irrelevant based on our DNA replication stress hypothesis. We then focused on candidates aligned with the hypothesis and had sufficient peptide spectrum matches (PSMs). Among the proteins considered, MSH6, MCM5, and RRM1 met our criteria for further validation (PSM counts 35, 40, and 29, respectively) (**Figure R3A**). We examined these candidates through immunoblotting, where only RRM1 showed a significant and reproducible reduction in protein levels in HEK293T-DPH4KO cells (**Figure 6D and R4**). We thus prioritized RRM1 in our study.

The reviewer's question prompted us to revisit the SILAC raw data and consult with proteomics experts to address why MSH6 and MCM5, which showed decent H/L ratios and *p*-values, were false positives. We refined our data analysis approach using updated software (Proteome Discoverer 3.0) and the latest Uniprot database. We switched to 'nested design' for data processing and 'abundances by bio-replicates' for H/L ratio calculation. Details of the revised data processing procedures are provided in the **Methods** section. This updated data analysis method, reflected in the new volcano plot (**Figure R3B**), showed that the revised MCM5's H/L ratio is approximately 0.85, consistent with our immunoblotting results (**Figure R4**). For MSH6, we manually checked all detected peptides and found that most showed a H/L ratio between 0.8 and 0.9, matching our immunoblotting results. However, one outlier peptide with H/L ratio of 0.026 and a very low *p*-value and high PSMs was detected. Since we cannot exclude this peptide for quantification due to its high confidence, the overall H/L ratio of MSH6 was significantly skewed by this peptide.

The revised analysis did not significantly alter the H/L ratio and *p*-value for RRM1 (**Figure R3A and R3B**) or most other proteins. It also did not introduce new reliable candidates relevant to DNA replication stress. To further validate the SILAC results obtained with the new data analysis methodology, we tested two additional candidates, IDH2 and SPAG9 (**Figure R3B**, highlighted in orange). IDH2 has a H/L ratio and *p*-value similar to RRM1, and SPAG9 was exclusively detected in the 'light samples' (WT cells). Immunoblotting confirmed that IDH2 and SPAG9 indeed exhibited decreased protein levels in HEK293T-DPH4KO cells (**Figure R5**), aligning with our proteomics results.

We have updated the manuscript and supplementary materials with the new volcano plot and the revised list of protein candidates.

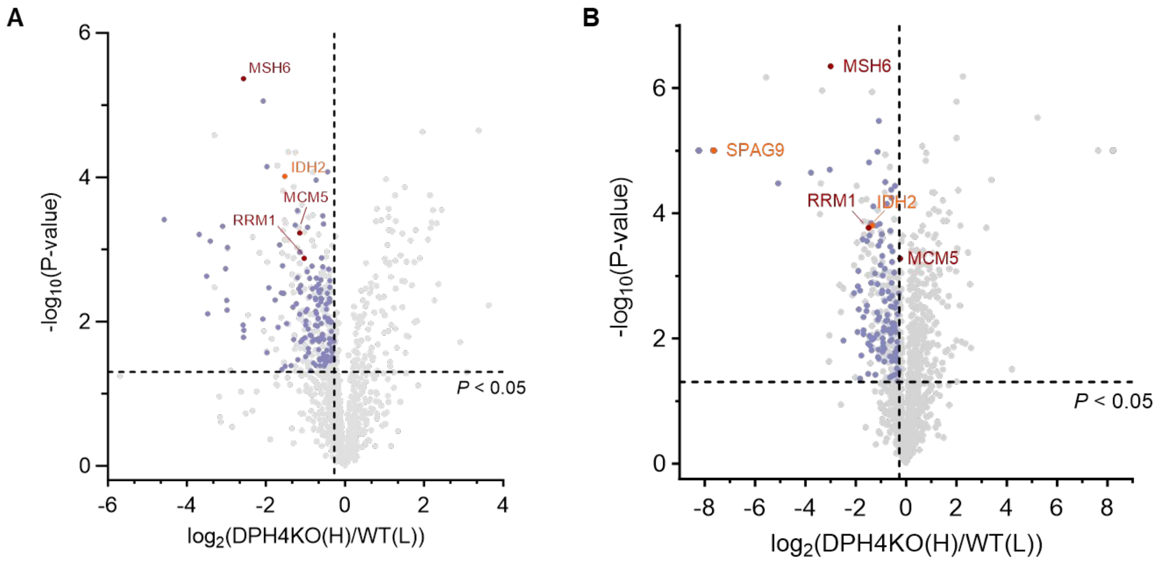

Figure R3. The original and revised volcano plot of the SILAC proteomics. (A) The original volcano plot in the manuscript with protein candidates related to DNA replication stress highlighted in dark red. MSH6, MCM5, and RRM1 are three protein candidates related to replication stress identified in the SILAC experiment with decent H/L ratios and *p*-values. **(B)** The revised volcano plot for SILAC proteomics. MSH6, MCM5, and RRM1 were labeled in dark red. Two additional chosen candidates, IDH2 and SPAG9, were labeled in orange.

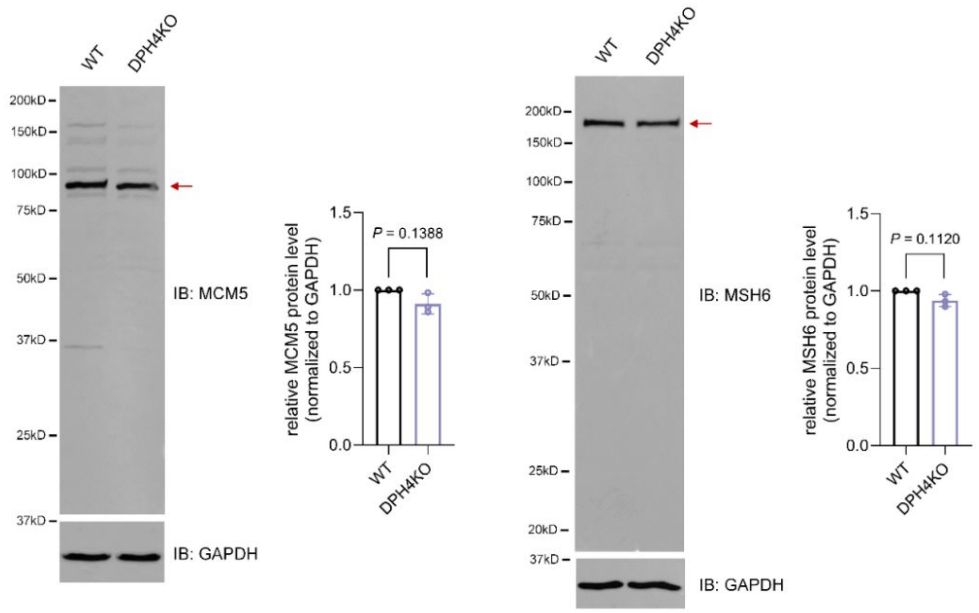

Figure R4. Immunoblotting verification of protein candidates related to DNA replication stress. MCM5 and MSH6 endogenous protein levels did not show significant difference between HEK293T-WT and DPH4KO cells. Representative data from three biologically independent experiments.

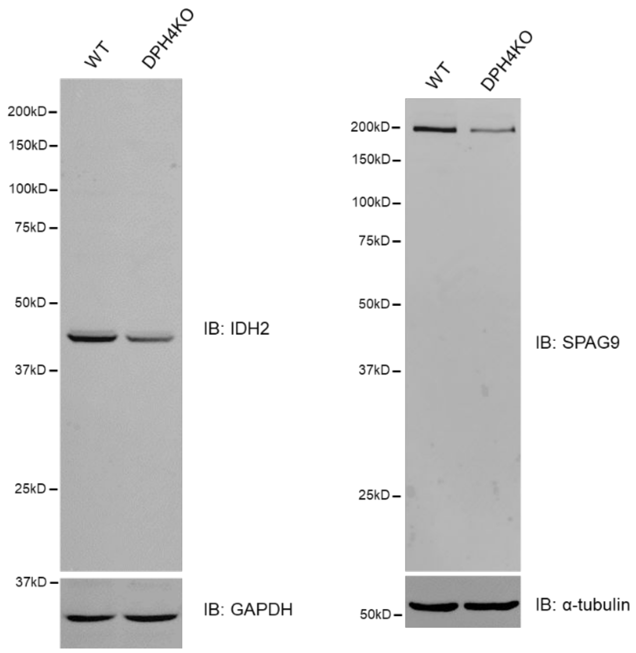

Figure R5. SPAG9 and IDH2 from the protein candidate list were verified through immunoblotting. IDH2 and SPAG9 exhibited decreased protein levels in HEK293T-DPH4KO cells. Representative data of two biological replicates.

4. Fig 6D shows only a small part of the blot with approx. 50% signal reduction for RRM1 protein. A frameshift generated smaller RRM1-E520stop fragment should also be detectable on the blot.
--> Please provide complete blots (suppl.data for all Westerns) and explain in case the truncated RRM1 protein is not detected.

The RRM1 antibody used in our study was obtained from CST (#8637), which was produced by "immunizing animals with a synthetic peptide corresponding to residues near the carboxy terminus of human RRM1 protein (792aa in total)". Therefore, this antibody is unable to detect the truncated RRM1 (amino acids 1-520). Please refer to **Figure R6** for the complete blot using this antibody to detect RRM1.

In response to the reviewer's comment, we explored whether other RRM1 antibodies may allow us to detect the truncated RRM1. As outlined in **Table R2**, most commercially available RRM1 antibodies also utilize the carboxy terminus of human RRM1 protein as the immunogen. We found one antibody from Santa Cruz (sc-377426) that utilizes amino acids 1-300 of human RRM1 as the immunogen. However, even with this antibody, we also failed to detect the truncated RRM1 (**Figure R6**). We attributed this to the fact that premature translational terminated proteins are prone to rapid degradation in cells (Lacsina, J. R. et al.), especially when the truncated proteins are unfolded and expose hydrophobic patches that serve as degradation signals (Huang, L. et al.). To investigate this further, we utilized *AlphaFold3* to predict the structure of truncated human RRM1 (1-520) and aligned it to the reported human RRM1 structure. This analysis revealed exposure of hydrophobic patches on protein surfaces in all five predicted models compared to full-length RRM1 (**Figure R7**).

Despite the challenges in directly detecting the truncated RRM1 protein via immunoblotting, our study provides robust supporting evidence from other assays, including RT-qPCR (**Figure 6E**), protein half-life (**Figure 6F**), and -1 frameshifting reporter assay (**Figure R8** below) for our conclusion that the observed reduced RRM1 protein level is attributed to -1 frameshifting during translation.

We have included the complete blots for all Westerns in the revised supporting materials (**Figure S6-S12**).

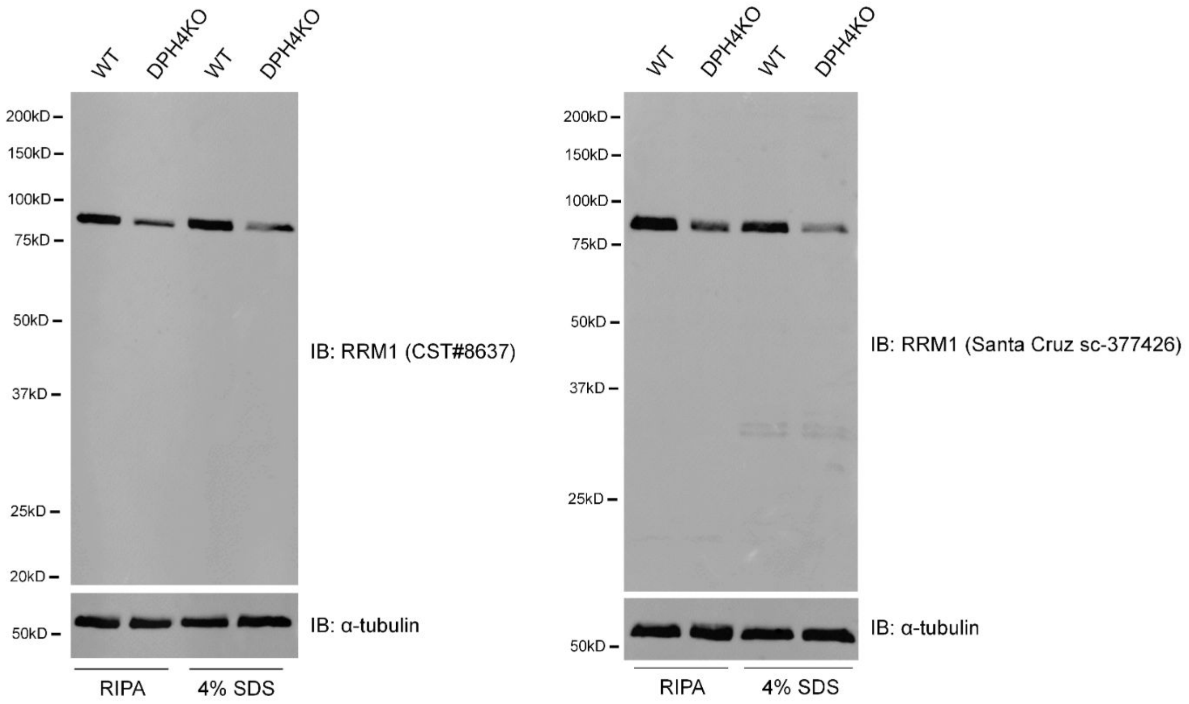

Figure R6. Complete blots of RRM1 with antibodies from CST and Santa Cruz. HEK293T-WT and DPH4KO cells were lysed in either RIPA or 4%SDS lysis buffer and resolved by SDS-PAGE. Antibodies for RRM1 from CST or Santa Cruz were used for the Western blot analysis.

Table R2. Immunogen comparison of the RRM1 antibody from different vendors.

Vendor	Catalog#	Immunogen
CST	8637	synthetic peptide corresponding to residues near the carboxy terminus of human RRM1 protein
CST	3388	synthetic peptide corresponding to residues near the carboxy terminus of human RRM1
Proteintech	10526-1-AP	amino acid 593-792 of human RRM1
Proteintech	60073-2-Ig	amino acid 593-792 of human RRM1
Thermo Fisher	MA5-26497	amino acid 541-792 of human RRM1
Santa Cruz	sc-377426	amino acid 1-300 of human RRM1

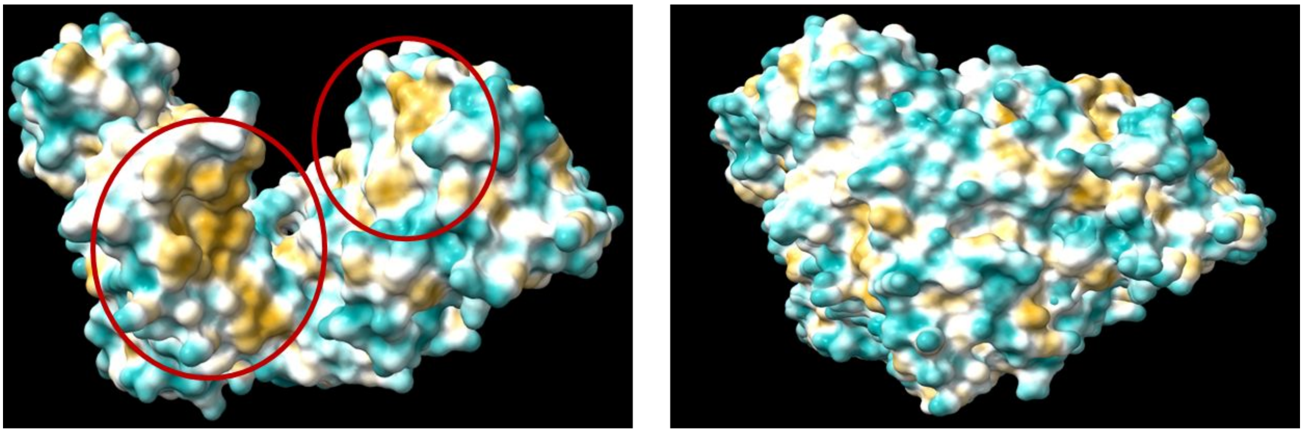

Truncated RRM1 (amino acids 1-520) Full-length RRM1 (aligned to left)

Figure R7. Structural alignment of the truncated RRM1 and full-length RRM1. The structure of truncated human

RRM1 (amino acids 1-520) was predicated by *AlphaFold3* and aligned to the structure of reported full-length human RRM1 (PDB ID: 6AUI). The hydrophobicity of protein surfaces was analyzed in *ChimeraX*, with cyan (most hydrophilic) to white to goldenrod (most hydrophobic). Hydrophobic patches on truncated RRM1 protein surface were highlighted in red circles. Representative data from one model was shown.

References:

Lacsina, J. R. et al. Premature Translational Termination Products Are Rapidly Degraded Substrates for MHC Class I Presentation. *PLOS ONE* 7, e51968 (2012).

Huang, L. et al. Hydrophobicity as a driver of MHC class I antigen processing. *EMBO J* 30: 1634–44 (2011).

5. Standard to assess -1 frameshift regions are dual reporter assays in the different reading frames.

--> Such analyses should be performed for the RRM1 region (incl. suggested stemloop/knot) to support the frameshift conclusions and to give some indication as for the level of frameshift.

In response to the reviewer's suggestion, we have incorporated the slippery sequence (CCCUUUU) and the adjacent 100 nucleotide stem-loop region (nt 1754-1873) of human RRM1 mRNA in a dual-luciferase reporter system (**Figure R8**). Since -1 frameshifting introduces a stop codon right after the slippery sequence, leading to immediate translation termination, we anticipated a decrease in the Firefly luciferase (FLuc) relative to *Renilla* luciferase (RLuc) activity in diphthamide-deficient cells. Our results confirm this expectation: the reporter system exhibited a significant decrease in the FLuc/RLuc ratio in DPH4KO cells (**Figure R8**), supporting our conclusion that diphthamide affects -1 frameshifting during RRM1 translation.

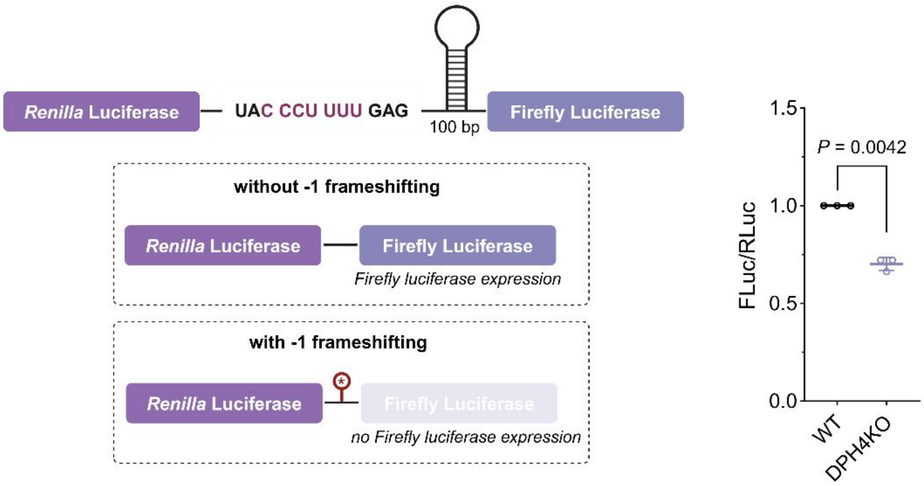

Figure R8. The designed dual-luciferase reporter system for evaluating RRM1 -1 frameshifting. The slippery sequence "CCCUUUU" and the adjacent 100 nt stem-loop region of human RRM1 mRNA were inserted between *Renilla* and Firefly luciferases. -1 frameshifting during translation is expected to decrease Firefly luciferase expression. Data with error bars are mean ± s.d. *P* value is determined using unpaired Welch's t-test. This figure was added to the revised manuscript as **Figure 6G**.

6. It was previously shown that diphthamide-deficient cells carry elevated ROS levels. Is it possible that ROS is the initial trigger of replication stress and RRM1 follows thereafter?

--> please explain

To investigate whether elevated ROS levels contribute to DNA replication stress in diphthamide-deficient cells, we treated HEK293T-WT and DPH4KO cells with the ROS scavenger *N*-acetylcysteine (NAC). We observed that up to 1 mM NAC treatment did not significantly change phosphorylated RPA32 (p-RPA32-T21) levels. However, we did observe a decrease in p-RPA32(T21) levels with 5 mM NAC treatment, while DPH4KO cells still bear a significantly higher p-RPA32(T21) level than WT cells (**Figure R9**). The fact that even under high-concentration NAC treatment, DPH4KO cells still have higher replication stress supports

that the decreased RRM1 protein level contributes to the replication stress, even though ROS may also contribute to DNA replication stress in diphthamide-deficient cells.

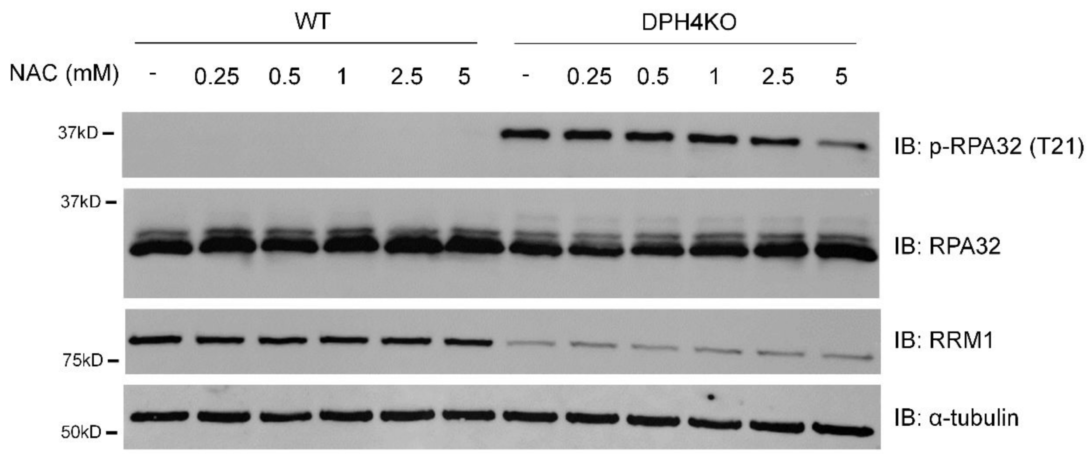

Figure R9. Assessment of p-RPA32(T21) levels in HEK293T-WT and DPH4KO cells upon *N*-acetylcysteine (NAC) treatment. HEK293T-WT and DPH4KO cells were treated with NAC at corresponding concentrations at 37 ^{0}C for 3h, and the p-RPA32 (T21) levels were assessed as an indication of DNA replication stress levels. Representative data of two biological replicates.

7. RRM1 is highly conserved from yeast to human.

--> Because the majority of 'biology-related' diphthamide analyses are/were done in yeast, please state if the proposed RRM1 frameshift feature also exist in yeast.

The yeast homolog of RRM1 is RNR1 (YER070W), which shows 59% sequence similarity to human RRM1 at the mRNA level. We found that the slippery sequence associated with -1 frameshifting in human RRM1 is absent in yeast RNR1. Instead, RNR1 has two alternative potential -1 frameshifting signals: 'AAAAAAC' (CDS 1248-1254) and 'UUUAAAC' (CDS 1440-1446), with minimal free energy (MFE) values of -15.5 and -16.4 kcal/mol, respectively. The -1 frameshifting at these sites would result in premature stop codons, leading to truncated proteins. Therefore, we sought to compare endogenous yeast RNR1 protein levels in normal and diphthamide-deficient strains as an indirect method to assess RNR1 -1 frameshifting events.

Due to the lack of antibodies for yeast RNR1 protein, we tagged the C-terminal of RNR1 with a 3xFlag tag at the endogenous loci using homologous recombination in BY4741 WT and diphthamide-deficient strains (**Figure R10A**). We then assessed RNR1 levels using Flag signals in immunoblotting. Our results indicated no significant difference in RNR1 protein levels between WT and diphthamide-deficient strains (**Figure R10B**), indicating that RNR1 translation is not affected by diphthamide under the tested conditions.

Even though the protein function of yeast RNR1 and human RRM1 is conserved, their sequence similarity at the mRNA level is relatively low compared to higher mammals (pig-92%, cattle-93%, and monkey-98%). This discrepancy may account for the differences in -1 frameshifting modulation between the two organisms.

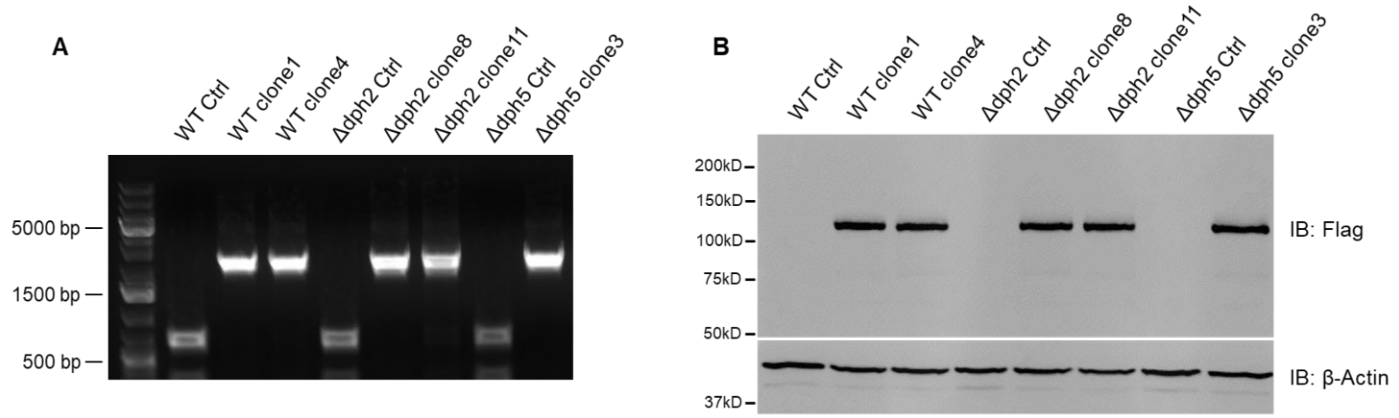

Figure R10. Examine endogenous RNR1 levels in normal and diphthamide-deficient yeast strains. (A) Endogenous RNR1 in yeast BY4741 WT, Δdph2, and Δdph5 strains was tagged with a C-terminal 3XFlag tag via genome editing. Primers from 500 bp before (forward) and 100 bp after (reverse) the RNR1 stop codon were used to amplify yeast genomic DNA. A shift from ~600bp to ~2200bp (addition of 3xFlag and the HIS3MX6 selection marker) was observed in the knocked-in strains. The PCR products were purified and further confirmed through sequencing. **(B)** Yeast cells were cultured in YPD broth to OD_{600} = 0.6. Total protein was extracted via NaOH-trichloroacetic acid precipitation. Endogenous RNR1 protein levels were assessed through immunoblotting. Representative data of two biological replicates. This figure was added to the supporting materials as **Figure S5**.

8. Title: "Diphthamide regulates mammalian DNA replication stress by modulating the translation of RRM1"
--> The data do not support physiological 'regulation' I suggest to stick to the data like Loss of diphthamide affects the translation of RRM1 and causes DNA replication stress in HEK293T cells.

We appreciate the reviewer's feedback on our manuscript's title. We have changed the title to "Loss of diphthamide increases DNA replication stress in mammalian cells by modulating the translation of RRM1".

9. Introduction: Despite its chemically challenging biosynthetic pathway, diphthamide is exclusively found on one protein eEF2, and evolutionarily conserved in all eukaryotes.
--> Narrowe, et al 2018 provide evidence for absence of diphthamide in eukaryotic parabasilids. Please correct 'all eukaryotes'.

We acknowledge the reviewer for bringing this to our attention. We have revised the statement in the manuscript to 'most eukaryotes' and included the reference from Narrowe et al. (2018).

10. Abstract: Employing a novel strategy, we identified diphthamide-regulating protein candidates by combing computational profiling of the human transcriptome and quantitative proteomics.
--> I guess they mean diphthamide regulated candidates as there is no evidence that those proteins regulate the synthesis of diphthamide. And (see comment to title) 'regulation' may not be appropriate (better diphthamide- dependent/modulated/affected throughout the manuscript). 'Combing': please check for and correct typos throughout the manuscript.

We have corrected all the typos and changed the term 'diphthamide-regulating' to 'diphthamide-affected'. We have also revised other parts of the manuscript to use terms such as 'affected' or 'modulated'.

11. Abstract & Discussion: 'Our results provide explanations for the association between diphthamide deficiency and cancer'; …. our results provide a logical explanation for why diphthamide deficiency promotes tumors…'
--> Because several other proteins are modulated by presence/absence of diphthamide, incl. some proteins

that appear to be strongly increased in DPHko cells (37kD & 15kD see Fig. 5A), I suggest to tone down conclusions that are related to RRM1 and cancer.

We have revised the Abstract and Discussion as suggested. Specifically, we changed '…provide explanations for the…' to '…provide a *potential* explanation for the…,' and '…our results provide a logical explanation for…' to '…our results provide a *potential* explanation for…'. Our intention was to suggest that our findings could offer one possible explanation, or an additional molecular mechanism, for the association between diphthamide deficiency and cancer rather than attributing the role of diphthamide in cancer solely to our results. We acknowledge that other proteins modulated by diphthamide deficiency may also contribute to this association. We have clarified this point in the revised manuscript to ensure that our conclusions are presented with appropriate caution.

oc-2024-00967k.R2

Name: Peer Review Information for "Loss of diphthamide increases DNA replication stress in mammalian cells by modulating the translation of RRM1"

Second Round of Reviewer Comments

Reviewer: 1

Comments to the Author
The authors addressed all topics in a very thorough and comprehensive manner - great paper.

Author's Response to Peer Review Comments:

Dear Editor,
Thanks for much for the great news! We have revised the SI as directed. Please let me know if you need any further revision from us.
Best regards,
Hening
